# Polyacrylonitrile-Based Fibrous Sorbents for Sorption–Spectroscopic Determination of Class I–III Toxic Metal Ions: Structure, Functionalisation, and Analytical Performance

**DOI:** 10.3390/polym18182218

**Published:** 2026-09-11

**Authors:** Aisha Nurlybayeva, Dinara Omarova, Zulaykho Smanova, Gaukhar Tazhkenova, Ainur Seitkan, Gulsim Matniyazova, Damen Nurgaliyeva, Bekzat Saurbayeva, Zulfiya Unerbayeva, Myrzabek Yermakhanov, Bakytgul Kussainova

**Affiliations:** 1 Department of Chemistry and Chemical Technology, Faculty of Technology, M.Kh. Dulaty Taraz University, Taraz 080012, Kazakhstan; rustem_ergali@mail.ru; 2 Department of Analytical Chemistry, Faculty of Chemistry, National University of Uzbekistan, Tashkent 100174, Uzbekistan; smanova.chem@mail.ru; 3 Department of Chemistry, Institute of Natural Sciences, L.N. Gumilyov Eurasian National University, Astana 010008, Kazakhstan; gaukhar-1970@mail.ru; 4 Department of Environmental Management and Engineering, L.N. Gumilyov Eurasian National University, Astana 010008, Kazakhstan; 5 Higher School of Natural Sciences, Astana International University, Astana 010008, Kazakhstan; gulsim.matniyazova@mail.ru (G.M.); nurgalieva0109@bk.ru (D.N.); 6 School of International Engineering, D. Serikbayev East Kazakhstan Technical University, Ust-Kamenogorsk 070004, Kazakhstan; saurbaeva71@mail.ru; 7 Department of Chemistry, Faculty of Natural Sciences and Geography, Abai Kazakh National Pedagogical University, Almaty 050010, Kazakhstan; uner_68@mail.ru; 8 Department of Chemistry, Higher School of Natural Sciences and Pedagogy, M. Auezov South Kazakhstan University, Shymkent 160012, Kazakhstan; myrza1964@mail.ru; 9 Faculty of Natural Sciences and Geography, M. Utemisov West Kazakhstan University, Uralsk 090000, Kazakhstan; nurbol-bakytgul@mail.ru

**Keywords:** polyacrylonitrile, fibrous sorbents, sorption–spectroscopic analysis, toxic metal ions, reagent immobilisation, diffuse reflectance, chelating fibres, green analytical chemistry

## Abstract

Fibrous polyacrylonitrile (PAN) is a versatile platform for solid-phase analytical chemistry because of its chemically transformable nitrile groups, mechanical robust-ness, and favourable fibre morphology. This review critically examines PAN-based fi-brous sorbents for sorption–spectroscopic analysis (SSA) of toxic metal ions, focusing on polyamine-modified PAN/polyethylenepolyamine/1,2-dichloroethane (PPD) and PAN/polyethylenepolyamine/acrylonitrile (PPA) matrices, iminodiacetate-containing fibrous ion-exchange (FIBAN) fibres, triethanolamine-modified PAN (PAN-T), and electrospun amidoximated PAN nanofibres (AOPAN). Relationships among polymer composition, fibre morphology, surface functionalisation, reagent immobilisation, ion transport, and optical response are evaluated with respect to sensitivity, selectivity, stability, reuse, and practical applicability. Particular attention is given to electrostat-ic/physical versus covalent immobilisation, matrix interference, and the distinction between adsorption capacity and direct solid-phase optical performance. Representa-tive systems include PPD–Arsenazo III for Pb(II), with a minimum detectable concen-tration of 0.24 μg L^−1^, and AMADA immobilised on a polyeth-ylene-polyamine-modified PAN matrix for Mn(II), with a reported determination limit of 0.02 μg mL^−1^. The review also considers sustainability, automation, nanostructured architectures, additive manufacturing, and portable optical detection. Current evi-dence indicates that no single PAN architecture is universally superior; future progress requires improved sorbent standardisation, systematic interference and leaching stud-ies, reproducible optical calibration, and validation under realistic sample conditions. Fibre diameter and porosity are also discussed as coupled determinants of diffusion, scattering, and reproducibility.

## 1. Introduction

Among synthetic polymers evaluated as solid-phase carriers in analytical chemistry, polyacrylonitrile (PAN) occupies a distinctive position: its nitrile side group (–C≡N) is chemically reactive toward amination, amidoximation, and polyamine grafting, while the polymer backbone is drawn into mechanically robust, high-surface-area fibres by conventional or electrospinning routes [1,2,3,4]. This combination—a tunable surface chemistry on a fibre format—makes PAN-based materials attractive not only for separation and remediation but, as this review focuses on, as carriers for immobilised optical reagents in sorption–spectroscopic analysis (SSA) of toxic metal ions, where the fibre itself becomes the analytical read-out surface.

Toxic heavy-metal ions entering aquatic environments through industrial discharge, mining effluents, and ageing infrastructure accumulate in sediments and food chains over decades [5]. In Kazakhstan and Central Asia, this intersects with evolving water-resource governance [6,7] and adsorption-based remediation strategies [8,9], which complement the detection-oriented approach reviewed here. Under the Russian/CIS Hygienic Standards (GN 2.1.5.1315-03), harmonised with WHO [10], contaminants are ranked: Class I (Pb, Cd, Hg—IARC carcinogens, no safe threshold), Class II (Cu, Cr, Ni, Co—micronutrients toxic at industrial concentrations), and Class III (Mn, Zn—toxic only at chronic exposure) [11]. WHO (2022) MACs are 10 μg L^−1^ (Pb), 3 (Cd), 6 (Hg), 50 (total Cr), and 80 (Mn) [10]—at or below the 10–100 μg L^−1^ sensitivity limit of direct UV–Vis spectrophotometry, making pre-concentration analytically obligatory. Solid-phase sorbents for SSA fall into two categories. Natural biosorbents (orange peel, banana peel, rice husk, chitosan) offer low cost and circular-economy alignment but suffer batch variability and acid-matrix dissolution. Synthetic fibrous polymer sorbents—principally PAN-based matrices—offer defined surface chemistry, controlled ion-exchange capacity, and mechanical robustness, with optical compatibility for elution-free SSA. This review is restricted to the latter, with particular emphasis on the PPD (PAN/PEPA/dichloroethane) and PPA (PAN/PEPA/acrylonitrile) matrices developed at the National University of Uzbekistan; natural sorbents are outside its scope.

Flame and electrothermal AAS, and inductively coupled plasma mass spectrometry (ICP-MS), meet the sensitivity requirements but depend on costly instrumentation, multi-step acid digestion, and—when co-precipitation is used for pre-concentration—substantial organic waste and energy consumption [12]. The environmental burden of conventional pre-concentration can be substantial when procedures rely on multi-step sample preparation and organic solvents. The AGREE framework provides a quantitative basis for comparing such procedural impacts across analytical methods [13]. This gap between regulatory sensitivity demands and the sustainability requirements of modern analytical practice defines the central motivation for sorption–spectroscopic analysis on fibrous solid supports.

Sorption–spectroscopic analysis (SSA) addresses this gap by combining pre-concentration and optical detection in a single aqueous-phase step [14,15]. An organic chromogenic or fluorescent reagent is immobilised on a fibrous carrier; the sample contacts the disc or minicolumn; the target metal ion is selectively retained as a surface complex; and the analytical signal—absorbance (A), diffuse reflectance (R∞) quantified via the Kubelka–Munk function F(R∞) = (1 − R∞)2/(2R∞), or luminescence intensity (Ilum)—is read directly from the solid phase, eliminating the elution step entirely [15]. Reagent consumption is reduced to <5 mL aqueous buffer per determination, analysis time to 10–20 min, and organic waste to negligible quantities. Among the solid carriers evaluated for SSA, synthetic fibrous polyacrylonitrile (PAN) sorbents are particularly attractive: they are optically transparent in the 400–700 nm visible range, mechanically stable over repeated regeneration cycles, and carry reactive nitrile groups (–C≡N) that can be converted by reaction with polyethylenepolyamine (PEPA) into chelating amidine (–C(=NH)–NH_2_) and amine (–NH_2_) functionalities [16,17].

The PPD matrix (PAN/PEPA/1,2-dichloroethane) and PPA matrix (PAN/PEPA/acrylonitrile) developed at the National University of Uzbekistan are polyamine-rich PAN-derived supports in which protonated amino groups can retain anionic analytical reagents while nitrogen-donor sites can participate in metal–ion interactions [16,17]. The PPD–arsenazo III system achieves Cmin = 0.24 μg L^−1^ for Pb^2+^—80-fold below direct solution spectrophotometry under identical reagent conditions [18]. Despite these analytical merits, PPD and PPA matrices share limitations common to laboratory-prepared sorbents: analytical performance can depend on the degree of PAN modification and reagent loading, and no certified reference sorbent material currently exists for inter-laboratory standardisation. These limitations, alongside the absence of systematic green chemistry benchmarking for SSA procedures, motivate the present critical review.

Several reviews have addressed SPE of heavy metals with polymeric sorbents [19], SSA on granular phases [20], and green aspects of sample preparation [21]. No systematic review has focused specifically on fibrous PAN-based sorbents across all three optical-detection modes—absorbance, diffuse reflectance, and luminescence—while quantitatively benchmarking the green analytical profile of the reviewed procedures against competing pre-concentration strategies. The present work fills this gap.

The objective of this review is to determine how PAN backbone composition, fibre morphology, surface functionalisation, and reagent immobilisation jointly control the analytical performance of PAN-based sorption–spectroscopic analysis. Four questions guide the discussion: which PAN architectures support direct optical read-out; how electrostatic/physical and covalent immobilisation differ in reagent retention, leaching, stability, and reuse; how common matrix components alter selectivity; and which structural or manufacturing approaches can improve reproducibility and field deployment. Section 2 is restricted to PAN-derived fibrous platforms. Non-PAN materials are mentioned later only when they provide an explicit analytical benchmark that cannot be obtained from the PAN literature.

The review uses the recent 2015–2025 literature together with earlier foundational studies. An overview of the screened literature corpus, including annual publication trends, the historical publication timeline, and the frequency of explicit AGREE/GAPI reporting, is provided in Figure S1 (Supplementary Materials). Section 2 compares PAN fibre composition, morphology, and chemical modification; Section 3 focuses on reagent immobilisation chemistry; Section 4 compares metal-specific procedures; Section 5 evaluates real-matrix validation; and Section 6 links structure–property relationships to standardisation, automation, nanostructured architectures, additive manufacturing, and portable optical detection. This organisation separates material chemistry from analytical application and avoids repeating the same evidence across sections. The hazard classifications and regulatory drinking-water values relevant to the target metal ions are summarised in Table 1.

**Figure 1:**
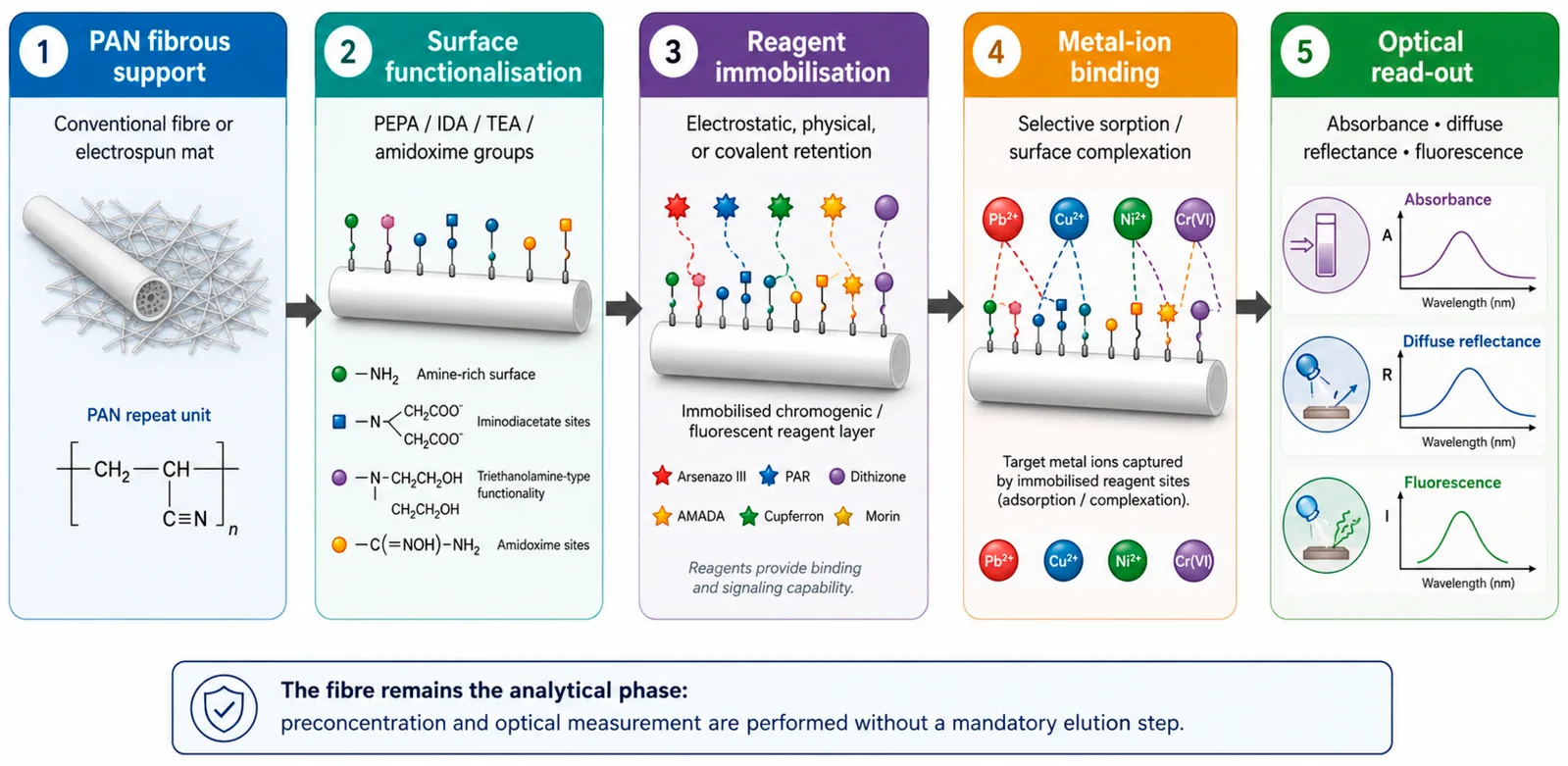
Simplified workflow for PAN-based sorption–spectroscopic analysis (SSA). A PAN fibrous support is chemically functionalised, loaded with a chromogenic or fluorescent reagent, contacted with the sample, and used directly as the optical measurement phase. Reagent retention may be electrostatic/physical or covalent; the target metal is then quantified by absorbance, diffuse reflectance, or fluorescence without a mandatory elution step. The figure is an author-reviewed scientific schematic prepared with AI-assisted visualisation software (Oreate, https://www.oreateai.com/).

The overall PAN-based SSA workflow used throughout this review is illustrated in Figure 1.

## 2. Polyacrylonitrile-Based Fibrous Sorbents: Structure and Functionalisation

The performance of a PAN-based SSA procedure depends on both the analytical reagent and the polymer carrier. For a direct comparison, the platforms below are evaluated using the same descriptors: backbone/fibre morphology, surface functionality, interaction mechanism, representative kinetics or analytical figure of merit, stability/reuse, and the principal limitation. Non-PAN foams, poly(methyl methacrylate), and mesoporous silica are excluded from this section because they do not share the polymer chemistry that defines the scope of the review.

### 2.1. PAN Backbone Composition and Fibre Architecture

PAN used for fibre production is not necessarily a homopolymer. High-acrylonitrile copolymers containing small fractions of comonomers such as itaconic acid or methyl acrylate are used to modify solution spinnability, chain packing, and subsequent structural development. Liu et al. compared electrospun homo-PAN, binary PAN (99 wt% acrylonitrile/1 wt% itaconic acid), and ternary PAN (92.8 wt% acrylonitrile/1.2 wt% itaconic acid/6 wt% methyl acrylate) and showed that composition measurably changed macromolecular regularity, orientation, and thermal behaviour [25]. Although that work addressed precursor nanofibres rather than SSA, it demonstrates that the starting polymer composition can alter the fibre structure before analytical functionalisation.

For PAN-based SSA, no homopolymer/copolymer composition has been demonstrated as universally optimal. A high acrylonitrile fraction preserves a high density of nitrile groups available for amination or amidoximation, whereas a small comonomer fraction may improve fibre formation and alter porosity, crystallinity, solvent accessibility, and mechanical integrity. The relevant selection criterion is therefore the combined effect of nitrile-group density and fibre morphology, not the polymer label alone. Future studies should report the actual comonomer composition of the PAN precursor whenever it is known.

Fibre diameter and porosity affect ion diffusion, equilibration time, and light transport through the sorbent layer. Electrospun submicrometre mats generally shorten diffusion paths and increase accessible surface area, but their higher porosity and refractive-index heterogeneity can also change diffuse scattering and the effective optical path. The available PAN–SSA literature does not define a universal optimum fibre diameter or porosity range. A smaller fibre is therefore not automatically analytically superior; morphology must be optimised together with reagent loading, calibration geometry, and mechanical stability [2]. The principal PAN-based fibrous sorbent platforms are compared in Table 2.

### 2.2. PPD and PPA Matrices—Polyamine-Functionalised PAN: Properties and Limitations

**Figure 2:**
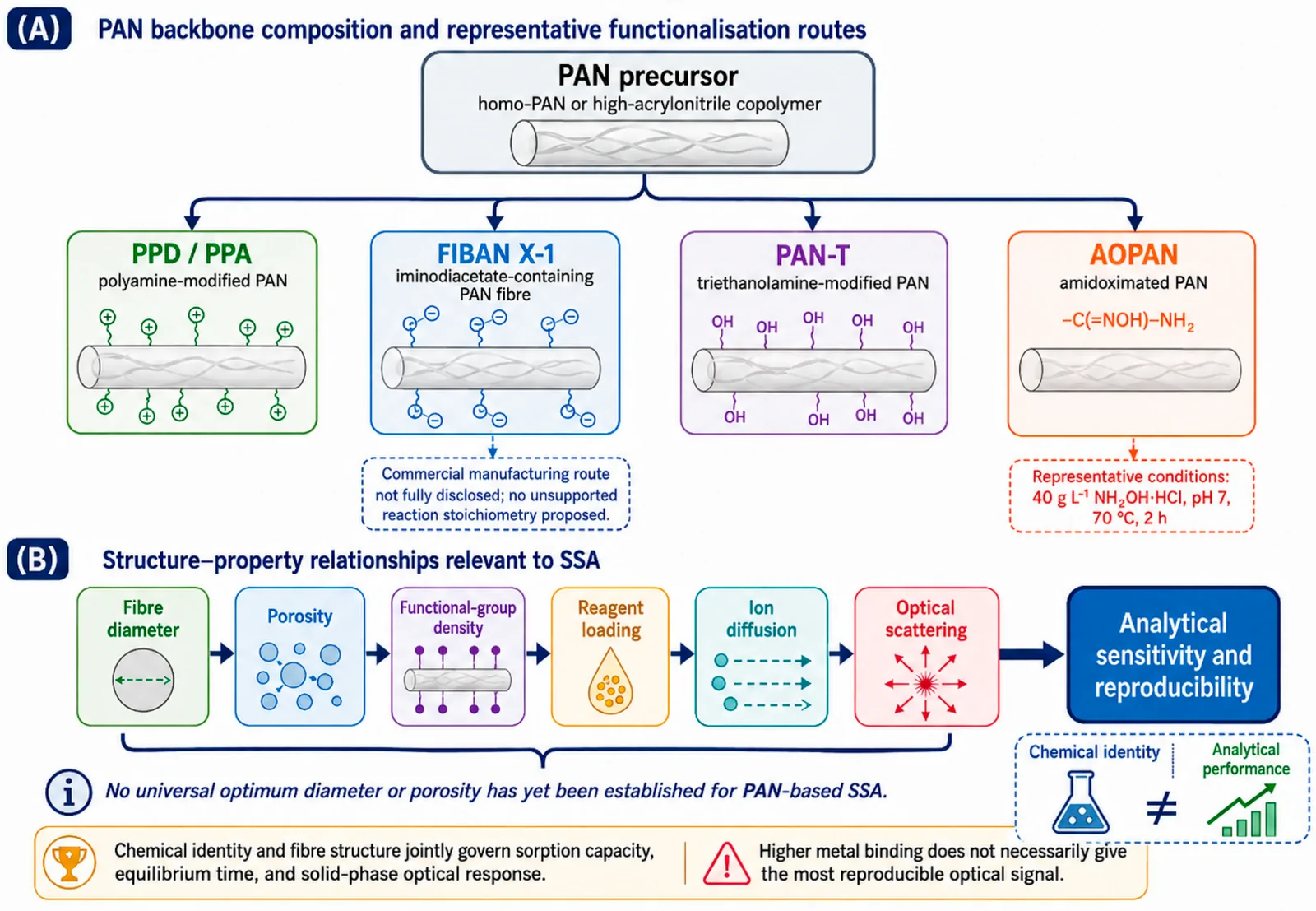
PAN backbone composition, representative functionalisation routes, and structure–property relationships relevant to SSA. (**A**) PAN may be used as homo-PAN or a high-acrylonitrile copolymer; the fibre is subsequently converted to polyamine-modified PPD/PPA adapted from [16,17], iminodiacetate-containing FIBAN X-1 adapted from [26,27], triethanolamine-modified PAN-T adapted from [28,33], or amidoximated PAN (AOPAN). The AOPAN pathway is shown with representative conditions of 40 g L^−1^ NH_2_OH·HCl, pH 7, 70 °C, 2 h adapted from [2]. The commercial manufacturing route of FIBAN X-1 is not fully disclosed, so no unsupported reaction stoichiometry is proposed. (**B**) Fibre diameter, porosity, functional-group density, reagent loading, ion diffusion, and optical scattering jointly determine analytical sensitivity and reproducibility; no universal optimum diameter or porosity has yet been established for PAN-based SSA adapted from [2,25]. The figure is an author-reviewed scientific schematic prepared with AI-assisted visualisation software (Oreate).

Polyamine-functionalised PAN does not form a single uniform repeat-unit product; modification produces a heterogeneous N-rich surface. A recent PPA preparation used sequential alkaline hydrolysis, activation/amination with hydroxylamine hydrochloride and hydrazinium chloride, grafting with polyethylenepolyamine (PEPA), and acid activation [16]. The practical chemical consequence is partial conversion of PAN nitrile groups into amide/amidoxime/amine- and amidine-like functionalities that can retain analytical reagents and participate in metal–ion binding. Because the degree of conversion depends on the preparation protocol, the reaction is represented in Figure 2 as a surface-functionalisation pathway rather than as a single stoichiometric polymer product.

The analytical performance of PPD/PPA-type materials is related to the simultaneous presence of reagent-retention and metal-binding sites. Protonated amino groups can facilitate the retention of anionic analytical reagents through electrostatic interactions, whereas nitrogen donor groups can participate in metal–ion binding under appropriate experimental conditions [30,31]. This combination allows reagent immobilisation and metal–ion interaction to occur on the same polymeric support. More generally, sorption–spectroscopic methods exploit the concentration of metal complexes on a solid phase and subsequent measurement of the analytical signal directly on, or from, the solid phase [31].

An important advantage of fibrous supports in appropriate sorption–spectrometric procedures is the possibility of performing the analytical measurement on the solid phase, thereby avoiding a separate elution step. This can simplify sample preparation and reduce the consumption of reagents and solvents compared with conventional liquid-phase extraction procedures [31].

Despite these advantages, PPD- and PPA-type matrices have several limitations that may constrain their broader inter-laboratory adoption. The chemical modification of PAN with polyethylenepolyamine produces a heterogeneous surface containing multiple nitrogen-containing functional groups, and the resulting sorption behaviour depends on the degree of polymer modification, reagent loading, solution pH, and the nature of the target metal ion [16]. Consequently, the preparation procedure and experimental conditions need to be carefully controlled when transferring an SSA method between laboratories. In addition, the analytical response of solid-phase spectrometric systems depends on the optical properties of the support and on the concentration of the immobilised reagent. For diffuse-reflectance measurements, the Kubelka–Munk function is commonly used to relate diffuse reflectance to the optical properties and surface concentration of the analyte; however, quantitative interpretation can be affected by scattering and sample properties [32]. Therefore, calibration conditions and reagent loading should be optimised for each analyte–carrier system. Finally, the absence of widely accepted certified reference materials specifically designed for PPD/PPA sorbents remains a practical obstacle to harmonised inter-laboratory comparison. Standardised preparation, characterisation, and validation procedures are therefore important for the further development of SSA methods based on fibrous PAN supports.

### 2.3. FIBAN Chelating Fibrous Ion Exchangers

The FIBAN series comprises fibrous ion-exchange materials developed in Belarus for the sorption and separation of metal ions from aqueous media. Among these materials, FIBAN X-1 is an iminodiacetate-containing fibrous-chelating ion exchanger obtained by chemical modification of a polyacrylonitrile fibre [26]. Its acid–base properties, sorption kinetics, equilibrium behaviour, and selectivity toward Ca(II), Cu(II), Pb(II), Cd(II), Ni(II), and Mn(II) have been systematically investigated. The sorption rate is particularly high, with a reported half-process time of approximately 24 s. In a neutral medium, the experimentally established selectivity sequence was Cu(II) > Ni(II) > Pb(II) > Cd(II) > Mn(II) > Ca(II), while complete extraction of the investigated heavy-metal ions was observed at pH > 4 [26].

A related iminodiacetate fibrous-chelating exchanger, FIBAN XC-1, has also demonstrated rapid metal–ion sorption under dynamic conditions. The material operated at flow rates of up to 20 column volumes min^−1^ and provided removal efficiencies of 82% for Cu(II) and 65% for Pb(II) during water purification experiments. The exchanger could be regenerated and retained its sorption and mechanical properties during repeated sorption–regeneration cycles [27].

These characteristics make FIBAN-type fibres attractive candidates for rapid metal–ion separation and preconcentration. However, their analytical selectivity is strongly dependent on the functional group, solution pH, competing ions, and the particular metal–ion system under investigation [26,27]. Therefore, FIBAN sorbents should not be assigned a universal selectivity or detection limit; performance parameters should instead be reported for the specific fibre, analyte, matrix, and analytical configuration employed.

### 2.4. Triethanolamine-Modified PAN (PAN-T) for Anion-Exchange SSA

Triethanolamine modification converts PAN into a weak-base anion-exchange fibre containing tertiary-amine and hydroxyl-rich surface functionality. Related synthesis work from the same research group showed that PAN–triethanolamine modification is favoured at approximately 175–180 °C; a 3 h treatment gave high exchange capacity, while longer treatment at 175 °C further increased the static exchange capacity [33]. The Cr(VI)-oriented PAN-T material reported subsequently had a pKa of 8.11 and an ion-exchange capacity of 2.03 meq g^−1^. At pH 2.8–3.1, protonation of the weak-base sites promotes retention of chromate/dichromate species, and the material retained about 94% of its initial capacity after ten sorption–desorption cycles [28].

For sorption–spectrophotometric determination of Cr(VI), 1,5-diphenylcarbazide can be used as the chromogenic reagent on a polyacrylonitrile-fibre-based solid support. This approach illustrates that PAN-based fibrous phases can combine Cr(VI) preconcentration with subsequent optical detection on the solid phase [34].

The strong dependence of Cr(VI) sorption on solution conditions is an important consideration for analytical applications of PAN-T. The optimised acidic pH range reported for Cr(VI) uptake reflects the protonation state of the weakly basic groups and the speciation of chromium in aqueous solution [28]. Consequently, PAN-T is particularly promising for the selective preconcentration and removal of Cr(VI), whereas its application to other metal ions would require separate optimisation of pH, competing ions, and the analytical detection scheme. Further work is therefore needed to establish PAN-T-based sorption–spectroscopic procedures for simultaneous or multi-element determination.

### 2.5. Electrospun PAN Nanofibre Membranes—Enhanced Surface Area and New Selectivities

Electrospinning produces non-woven PAN mats with submicrometre fibre diameters and high accessible surface area, providing short diffusion paths for subsequent functionalisation. A well-characterised amidoximation route converts surface nitrile groups to amidoxime groups using hydroxylamine. In the representative study used here, PAN nanofibres were treated with 40 g L^−1^ hydroxylamine hydrochloride at pH 7 and 70 °C for 2 h, conditions that produced substantial nitrile conversion while preserving a recognisable fibrous morphology [2]. The resulting amidoxime N/O donor sites increase metal–ion affinity, but the same nanoscale morphology can alter optical scattering; adsorption capacity and direct SSA sensitivity should therefore be evaluated separately.

Electrospun PAN fibres can also be modified with inorganic or chemical activating agents to alter their affinity toward individual metal ions. NaHCO_3_-activated PAN nanofibres (PANmod), for example, were investigated for the simultaneous adsorption of Pb(II) and Ni(II). The material exhibited a higher affinity for Ni(II) than for Pb(II), with the maximum adsorption performance observed at approximately pH 7.0. The study therefore demonstrates that post-treatment of PAN nanofibres can substantially modify their metal–ion sorption behaviour [35].

More complex hybrid architectures have also been developed by integrating porous functional materials with electrospun PAN membranes. For example, a triazine porous organic polymer based on cyanuric chloride and triphenylphosphine (CC-TPS) was grown in situ on PAN nanofibres to form PAN@CC-TPS membranes. The resulting material provided high enrichment factors for trace nitrophenol pollutants in water, illustrating the potential of electrospun PAN membranes as scaffolds for hierarchical functional coatings [36]. Although this system was developed for organic pollutants rather than metal ions, the strategy is relevant to the design of multifunctional PAN-based analytical membranes.

Another approach involves incorporating aminated polymeric nanospheres into electrospun PAN fibres. PAN/APN nanofibres exhibited a Cr(VI) adsorption capacity of 556 mg g^−1^ at 298 K. The adsorption kinetics followed a pseudo-second-order model, while mechanistic analysis indicated contributions from electrostatic adsorption, redox transformation, and chelation. These results demonstrate how incorporation of additional nitrogen-containing functionalities can substantially increase the metal-binding capacity of electrospun PAN materials [37].

Electrospinning expands the PAN design space through post-functionalisation, composite formation, and surface coating. Most published electrospun PAN materials, however, were developed for adsorption, extraction, or membrane separation rather than direct SSA. Their analytical transferability must therefore be demonstrated by reagent-retention tests, optical calibration on the intact mat, interference studies, and mechanical testing under flow.

### 2.6. Practical Selection Criteria: Sensitivity, Time, Cost, and Portability

The current evidence does not identify a single PAN platform as best for every application. PPD/PPA and related PAN analytical fibres have the strongest direct SSA evidence, including sub-µg L^−1^ performance for selected analytes and direct absorbance/diffuse-reflectance read-out. FIBAN X-1 provides the fastest reported sorption kinetics among the platforms compared, but its use as a carrier for direct immobilised-reagent optical measurement is less developed. PAN-T is particularly strong for Cr(VI) under acidic conditions and offers documented reuse, whereas AOPAN provides high surface accessibility but still requires stronger validation of optical reproducibility.

Published studies rarely report monetary cost in a form that allows a rigorous cross-study comparison. Practical cost can therefore be discussed only qualitatively: conventional PPD/PPA-type fibres are compatible with standard UV–Vis or diffuse-reflectance instrumentation; commercial/specialised chelating fibres may increase material cost; electrospun AOPAN requires an electrospinning step but can be fabricated as thin disposable or replaceable mats. For field use, the most portable formats are those that combine a thin PAN disc with controlled reflectance or fluorescence imaging. Analysis time, selectivity, and portability should thus be reported together with LOD rather than treating sensitivity as the sole performance criterion.

Figure 2 summarises the principal chemical routes used to functionalise PAN and highlights the combined influence of fibre structure, interfacial chemistry, mass transport, and optical properties on analytical performance.

The revised scheme separates chemical identity from analytical performance. PPD/PPA, FIBAN, PAN-T, and AOPAN differ in functional-group chemistry, but they are also built on fibre structures with different transport and optical behaviour. This distinction is essential when comparing sorption capacity, equilibrium time, and LOD: a material that binds more metal is not necessarily the material that gives the most reproducible solid-phase optical signal.

## 3. Functionalisation of PAN-Based Fibres with Chromogenic and Fluorescent Reagents

The choice of chromogenic or fluorescent reagent controls spectral sensitivity, but the immobilisation mode determines whether that sensitivity can be maintained during sample contact and repeated use. In PAN-based SSA, the reagent may be held by electrostatic/physical interactions on a charged fibre surface or attached covalently to a pre-functionalised PAN support. These routes should be distinguished because they differ in preparation simplicity, leaching risk, accessibility of the ligand, and regeneration behaviour.

The reagent systems below are therefore compared using four common criteria: optical response; immobilisation mode and reagent retention; pH-dependent selectivity/interference behaviour; and stability/reuse. Where a study does not report long-term leaching or cycling data, that absence is stated rather than extrapolated from another reagent or support.

### 3.1. Immobilisation Modes: Leaching, Stability, and Reuse

Electrostatic or physical immobilisation is experimentally simple and can preserve the native chromophore because no new covalent bond is introduced into the reagent. In PPD/PPA-type fibres, protonated amine sites can retain anionic dyes by ion pairing while neighbouring nitrogen donors participate in metal binding. The limitation is that reagent retention is inherently sensitive to pH, ionic strength, competing anions, and washing conditions. Consequently, leaching must be measured for the actual fibre–reagent pair; a universal percentage loss cannot be assigned to physically immobilised PAN systems [30,31].

Covalent attachment generally provides stronger reagent retention but can change ligand accessibility or the local electronic environment. The clearest PAN example is PAR immobilised on aminated PAN through a Mannich-type reaction: the reported material retained a visible heavy-metal response, was reusable for more than 50 cycles, and showed photostability for more than 30 days under the study conditions [38]. These data support the use of covalent immobilisation when long operational life is required, while also showing why covalent and electrostatic systems should not be compared solely by solution-phase stability constants.

Operationally, the preferred immobilisation route is application-specific. A rapidly prepared electrostatic layer may be adequate for low-cost disposable discs, whereas a reusable sensor or flow-through cartridge benefits from covalent retention. Long-term stability should be reported as reagent loss, change in blank signal, change in calibration slope, and retained response after defined regeneration cycles rather than by a qualitative statement of ‘good stability’. Representative PAN-supported chromogenic and fluorescent reagent systems are compared in Table 3.

### 3.2. Arsenazo III—A Sensitive Reagent for Pb(II) Determination

Arsenazo III is a highly sensitive azo-arsonate chromogenic reagent that has been widely employed in spectrophotometric metal–ion determination because of its pronounced colour response and strong complex-forming ability. Its analytical behaviour is strongly dependent on the acidity of the medium and on the nature of the metal ion, since protonation and metal-complex formation influence the position and intensity of the absorption bands. In solid-phase analytical systems, immobilisation of Arsenazo III can additionally combine the optical response of the reagent with sorption-based enrichment of the analyte.

A recent study demonstrated the application of Arsenazo III immobilised on a polymer matrix composed of polyacrylonitrile (PAN), polyethylene polyamine (PEPA), and dichloroethane (PPD) for the sorption–spectrophotometric determination of Pb(II). The PPD matrix was used as the polymeric support for the immobilised reagent, providing a solid-phase analytical platform for direct determination of Pb(II) in water samples [18].

The analytical response of the immobilised system was strongly dependent on the acidity of the medium. In an acetate-buffer medium at pH 5.8, the Pb(II)–Arsenazo III system immobilised on the PPD matrix exhibited its maximum analytical signal at 640 nm. Under these conditions, the method showed a minimum detectable concentration (C_min_) of 0.24 μg L^−1^ and a relative standard deviation (Sr) of 0.06, indicating good analytical reproducibility. The calibration response obeyed the Beer–Lambert–Bouguer relationship over a Pb(II) concentration range of 0.04–2.4 μg mL^−1^.

The reported statistical evaluation further supports the reliability of the analytical procedure. The authors assessed the accuracy and reproducibility using Student’s *t*-test and Fisher’s *F*-test. The experimental values were reported as t_exp_ = 1.09 compared with ttab = 2.83, and F_exp_ = 2.52 compared with F_tab_ = 4.47, respectively. Because the experimental values were below the corresponding tabulated values, the authors concluded that the proposed procedure provided acceptable accuracy and reproducibility under the investigated conditions.

An important analytical feature of this system is the combination of reagent immobilisation and sorption-based determination. In conventional solution spectrophotometry, the analytical response is generated in a homogeneous solution, whereas in the PPD system the reagent and analyte are concentrated within a heterogeneous polymeric phase before optical measurement. Consequently, the high sensitivity of the immobilised system should not be interpreted simply as an increase in the intrinsic molar absorptivity of Arsenazo III. Instead, the analytical improvement is more appropriately associated with the solid-phase enrichment process, localisation of the reagent, and the favourable microenvironment created within the polymer matrix.

The 640-nm analytical maximum is particularly useful for solid-phase measurement because it provides a defined wavelength at which the Pb(II)-dependent optical response of the immobilised reagent can be monitored. The reported linear range of 0.04–2.4 μg mL^−1^ also demonstrates that the system is applicable not only to trace-level detection but to quantitative determination over a measurable working concentration interval. The combination of a C_min_ of 0.24 μg L^−1^, Sr = 0.06, and the reported statistical validation therefore identifies the PPD–Arsenazo III system as a promising example of PAN-based sorption–spectroscopic analysis for Pb(II) [18,43,44].

The study also reported successful application of the method to water samples, supporting its practical analytical relevance. Nevertheless, the available primary publication does not provide sufficient evidence to assign a specific number of regeneration cycles, long-term storage lifetime, or photostability to the PPD–Arsenazo III material. These characteristics should therefore not be inferred from solution-phase studies of Arsenazo III or from stability data obtained for other immobilised reagents. Similarly, a solution-phase molar absorptivity should not be transferred directly to the immobilised polymer system because the optical geometry, effective path length, reagent loading, and local chemical environment are fundamentally different.

Overall, the PPD–Arsenazo III system represents a relevant example of how PAN-derived polymer matrices can transform a conventional chromogenic reagent into a solid-phase analytical material. The combination of immobilised Arsenazo III, polymer-supported sorption, a defined analytical wavelength of 640 nm, and a reported minimum detectable concentration of 0.24 μg L^−1^ demonstrates the potential of this approach for sensitive Pb(II) determination. Further studies addressing regeneration, storage stability, photostability, and broader matrix effects would nevertheless be valuable for assessing the long-term robustness of the material in routine analytical applications.

### 3.3. 4-(2-Pyridylazo)resorcinol (PAR)—pH-Staged Multi-Metal Selectivity

4-(2-Pyridylazo)resorcinol (PAR; C_11_H_9_N_3_O_2_) is a widely used azo chromogenic reagent for spectrophotometric determination of transition and heavy-metal ions. Its analytical behaviour is governed by the nitrogen and oxygen donor sites of the ligand and by the protonation state of the reagent, making the composition and stability of PAR–metal complexes strongly dependent on solution conditions. Quantitative studies of PAR complexation have established metal-dependent stability constants and molar absorption coefficients under controlled ionic strength and temperature, providing a useful reference for interpreting PAR-based analytical systems [45].

Kocyła et al. [45] systematically re-evaluated the molar absorption coefficients and stability constants of PAR complexes using spectrophotometric measurements under defined solution conditions. Their results demonstrate that the magnitude of the analytical response and the apparent stability of PAR complexes vary substantially among metal ions. Such solution-phase data are useful for understanding the coordination chemistry of PAR, but they should not be directly transferred to immobilised systems because immobilisation changes the local chemical environment, reagent accessibility, surface loading, and optical measurement geometry [45].

A particularly relevant example for PAN-based solid-phase analysis was reported by Li et al. [38], who prepared a colourimetric fibre by first aminating commercial polyacrylonitrile (PAN) fibre with ethylenediamine and subsequently immobilising PAR on the modified fibre through a Mannich reaction [38]. The resulting PAR–PAN fibre exhibited a visible colour response to Hg(II), Pb(II), Cd(II), Zn(II), Ni(II), and Cu(II) in neutral aqueous solutions, changing from red–orange to dark brown. In contrast, no comparable colour change was observed for Ca(II), Mg(II), or Al(III), demonstrating useful discrimination between heavy-metal ions and several common light-metal ions [38].

The PAN-based study is particularly important because it demonstrates that PAR can be converted from a conventional solution reagent into a covalently immobilised chromogenic fibre while retaining its ability to respond optically to several heavy-metal ions. The immobilisation was characterised using X-ray powder diffraction, scanning electron microscopy, FTIR, and UV–Vis spectroscopy, while the analytical material was further evaluated with respect to response rate, selectivity, reusability, photostability, and adsorption capacity [38].

The immobilisation chemistry is therefore a critical parameter in PAR-based sorption–spectroscopic analysis. Importantly, the PAN/PAR system reported by Li et al. used a Mannich-type covalent immobilisation route, rather than aryldiazonium coupling [38]. This distinction is important when comparing different polymer-supported PAR systems because the nature of the covalent linkage, surface functional-group density, and accessibility of the immobilised reagent can influence both the optical response and operational stability.

A different covalent immobilisation strategy has recently been demonstrated for PAR on Amberlite XAD-4, providing a useful non-PAN comparator. In that system, PAR was attached to the polymer through an aryldiazonium-mediated sequence involving surface arylation followed by conversion of surface nitro groups to anilines and subsequent diazotisation/azo coupling. The resulting XAD-4–PAR material was characterised by ATR-IR, TGA, and XPS and was evaluated for the extraction of Co(II), Ni(II), and Cu(II) from groundwater.

The XAD-4–PAR study demonstrates that aryldiazonium chemistry can provide an alternative route for covalent attachment of PAR to polymeric surfaces. However, because the support is a cross-linked polystyrene–divinylbenzene resin rather than PAN, the reported sorption properties and stability characteristics should be regarded as comparative evidence rather than direct evidence for PAN-based fibres. Extrapolation of this chemistry to PPD/PPA-modified PAN fibres therefore remains a prospective materials-development strategy rather than an experimentally demonstrated feature of the systems reviewed here [46].

Overall, the available evidence indicates that PAR is particularly attractive for PAN-based SSA because it combines broad heavy-metal responsiveness with the possibility of covalent immobilisation on functionalised PAN fibres. The comparison between the Mannich-functionalised PAN system and the more recent aryldiazonium-functionalised XAD-4 system also illustrates an important design principle: the immobilisation chemistry should be considered separately from the intrinsic solution-phase coordination chemistry of PAR. For PAN-based analytical fibres, future optimisation should therefore focus on controlling surface loading, reagent accessibility, regeneration behaviour, and matrix-specific selectivity rather than assuming that solution-phase stability constants alone predict solid-phase performance [46].

### 3.4. Dithizone and Thiocarbazone Systems—Soft-Metal Affinity and Stability Constraints

Dithizone (1,5-diphenylthiocarbazone, DTZ, HDz) is a classical sulfur- and nitrogen-containing chromogenic reagent widely used for the extraction and spectrophotometric determination of metal ions. Its analytical utility is associated with the formation of intensely coloured metal–dithizone complexes, while the relative response depends on the metal ion, solution acidity, ligand speciation, and competing complexation reactions. The strong affinity of dithizone for several heavy and transition metals has made it particularly relevant to solid-phase and membrane-based colourimetric systems.

A representative nanostructured application was reported by Takahashi et al., who prepared dithizone nanofibres and deposited them as a thin functional layer on a cellulose ester membrane for filtration-enrichment and colourimetric determination of Hg(II) [47]. The dithizone nanofibre layer was less than 500 nm thick and was formed uniformly on the membrane surface. Filtration of aqueous samples through the membrane resulted in enrichment of Hg(II) and a readily measurable colour change from steel blue to reddish brown. The membrane therefore combined analyte preconcentration with direct optical detection and provided a simple solid-phase format for trace Hg(II) analysis.

The performance of this system was evaluated under controlled filtration conditions. Quantitative Hg(II) extraction was achieved over a filtration flow-rate range of approximately 1.3–9.3 mL min^−1^, demonstrating that the fibrous layer could retain Hg(II) efficiently without requiring extremely slow sample processing. The authors also applied the membrane to artificial wastewater, certified river water, and seawater samples. For samples containing 10 μg L^−1^ Hg(II), recoveries were close to 100%, including 99% for artificial wastewater, 102% for certified river water, and 100% for seawater under the reported experimental conditions.

An important limitation of dithizone-based solid-phase systems is chemical stability. Dithizone and its nanofibres are susceptible to oxidative decomposition when exposed to air and illumination. In the membrane study, the colour brightness of freshly prepared dithizone membranes changed progressively during exposure, which the authors attributed to oxidative decomposition caused by air and/or light. This behaviour demonstrates that reagent stability must be considered when dithizone is incorporated into a fibrous analytical material [48].

The same study demonstrated that stability could be substantially improved by incorporating a reducing environment during nanofibre preparation and by limiting exposure to oxygen and light. Membranes prepared in the presence of ascorbic acid and subsequently stored in a sealed aluminium bag under vacuum with an oxygen absorber retained their original steel-blue colour for more than three months. Thus, the primary limitation is not an inherent lack of analytical sensitivity, but the susceptibility of the immobilised reagent to environmental oxidation and photochemical degradation.

Dithizone has also been incorporated directly into electrospun polymeric fibres. A polystyrene–dithizone (PS–DZ) nanofibre produced by co-electrospinning exhibited a dense fibrous network with fibre diameters of approximately 200–400 nm. In the reported application, the material was used as a solid-phase sorbent for Pb(II), and the functionalised nanofibre showed particularly strong retention of Pb(II). This work illustrates the potential of co-electrospinning as a straightforward strategy for incorporating dithizone into a polymeric fibrous matrix without requiring a separate surface-functionalisation step.

A different analytical application extends dithizone-based fibrous systems beyond the conventional soft-metal targets. Dewi et al. developed a dithizone-functionalised polyurethane electrospun nanofibre (DTZ/PU-NF) for selective colourimetric determination of Cr(VI). In this system, dithizone was first complexed with Co(II), and the resulting DTZ–Co(II) complex served as the recognition element. In the presence of Cr(VI), the analytical response was generated through a metal-exchange/redox process involving replacement of Co(II) by Cr(VI). The approach therefore differs fundamentally from conventional direct complexation of the analyte with free dithizone [49].

The DTZ–Co(II)/PU-NF material showed a mean fibre diameter of approximately 384 nm and produced a distinct colour response in the presence of Cr(VI). The colourimetric method exhibited a linear range of 0.001–1.0 mg L^−1^, with R^2^ = 0.990, a limit of detection of 0.001 mg L^−1^, and a reported precision of 1.7%. The authors further reported that the method remained selective in the presence of several potentially interfering metal ions and successfully applied the system to vegetable-oil samples.

Taken together, these studies demonstrate two complementary roles for dithizone in fibrous sorption–spectroscopic analysis. First, direct incorporation of dithizone into nanofibrous membranes can provide efficient enrichment and colourimetric detection of target heavy metals such as Hg(II). Second, pre-complexation of dithizone with a selected metal can be exploited to construct more selective reaction schemes, as demonstrated by the DTZ–Co(II)/PU-NF system for Cr(VI). At the same time, the documented sensitivity of dithizone to oxidation and illumination indicates that storage conditions, reagent formulation, and matrix environment are critical parameters for achieving reproducible performance [47].

Therefore, dithizone remains an attractive chromogenic component for fibrous solid-phase analytical systems, but its advantages should be balanced against its chemical stability. The literature supports the use of oxygen-restricted and antioxidant-containing preparation/storage conditions for improving stability, while further comparative studies are needed to determine how these effects translate to PAN-based fibres and other polymeric supports.

### 3.5. Xylenol Orange, DHNDSADNa, Cupferron, and AMADA—Complementary Selectivities

Xylenol Orange (XO) is a multidentate sulphonephthalein-based ligand widely used as a colourimetric complexing reagent for metal–ion determination. Its complexation behaviour depends strongly on the charge density and coordination characteristics of the metal ion. Belleza and Villaraza demonstrated that, under slightly acidic conditions (pH 5.8), XO forms complexes with Gd^3+^, Tb^3+^, Zn^2+^, Mn^2+^, and Ca^2+^, with substantially different detection responses. Gd–XO, Tb–XO, and Zn–XO complexes were detectable at submicromolar concentrations, whereas Mn^2+^ and Ca^2+^ required higher concentrations for observable complex formation. The authors attributed these differences primarily to the ionic charge density of the metal ions, demonstrating that XO can discriminate between metal ions with substantially different charge densities, although discrimination between ions such as Gd^3+^, Tb^3+^, and Zn^2+^ is more limited [50]. These findings are relevant to immobilised XO systems because the analytical response of the reagent is intrinsically dependent on the metal–ion coordination environment and the accessibility of the ligand donor groups.

4,5-Dihydroxynaphthalene-2,7-disulfonic acid disodium salt dihydrate (DHNDSADNa) provides a different analytical mechanism after immobilisation on a polyethylene-polyamine polyacrylonitrile (PPA) matrix. The immobilised reagent was developed for the selective determination of Cr(VI) and exhibited distinct spectroscopic bands at 460 nm for the reagent and 590 nm after interaction with Cr(VI). The reported detection limit was 2 μg mL^−1^, and the immobilised system showed selectivity in the presence of Co(II), Ni(II), Al(III), Fe(II), Ti(IV), Zr(IV), Ta(V), Mn(II), and W(VI) [41]. Thus, DHNDSADNa/PPA demonstrates the analytical advantage of combining reagent immobilisation with a pronounced spectral response to Cr(VI), although its reported detection limit and selectivity should be considered within the experimental conditions of the original study rather than generalised to all SSA matrices.

Cupferron and AMADA provide complementary examples of PAN-based sorption–spectrophotometric systems for Mn(II) determination. Smanova et al. reported the immobilisation of cupferron on a fibrous polyacrylonitrile-based PPD sorbent for Mn(II) determination in aqueous media [51]. The optimum conditions included an acidic medium, with the highest analytical response observed around pH 3, and the cupferron–Mn(II) system exhibited a maximum response at approximately 500 nm. The method showed a linear concentration range of 0.5–5.0 mg L^−1^ and was proposed for the determination of Mn(II) in industrial wastewater. The study also used X-ray diffraction, scanning electron microscopy, and infrared spectroscopy to investigate the immobilised system and the interaction of Mn(II) with the reagent-modified fibre.

AMADA (alizarin-3-methylamino-N,N-diacetic acid) represents another PAN-supported reagent system for Mn^2+^ determination. In contrast to the acidic cupferron system, AMADA is reported to operate under less-acidic conditions and provides a low detection limit for Mn^2+^. The AMADA/PAN system therefore expands the range of reagent chemistries available for manganese determination on fibrous polymeric supports [42]. Rather than assigning universal matrix-specific advantages to either reagent, these systems illustrate how the choice of immobilised chromogenic reagent can be used to adjust the working pH, spectral response, sensitivity, and selectivity of PAN-based SSA methods.

Overall, these four reagent systems illustrate complementary approaches to selectivity in immobilised sorption–spectrophotometric analysis. XO demonstrates the influence of metal–ion charge density on complex formation [50], DHNDSADNa provides a pronounced spectral response and selective Cr(VI) determination on a PPA matrix [41], while cupferron and AMADA demonstrate alternative PAN-supported approaches for Mn(II) determination under different chemical conditions [42,51]. Their analytical performance is therefore governed not only by the intrinsic coordination chemistry of the reagent but also by the chemical environment and accessibility created by immobilisation on the polymeric support.

### 3.6. Fluorescent Reagents and Computational Approaches—Extending Optical Sensitivity

Fluorescence-based sorption–spectroscopic approaches provide an alternative to absorbance measurements when the analytical response can benefit from the high sensitivity of fluorescence detection. An early example directly relevant to immobilised analytical reagents is the use of morin as a fluorescence sensor for Al(III). Saari and Seitz reported immobilised morin for fluorescence-based determination of Al(III), demonstrating that immobilisation of a chromophoric chelating reagent can be used to construct a solid-state optical sensing system [52]. The study provides an important precedent for incorporating fluorescent metal-binding reagents into analytical supports, although the characteristics of this early sensor should not be directly extrapolated to modern fibrous PAN-based SSA systems.

The fluorescence properties of the Al–morin system have also been investigated in solution. Hernandez and Medina Escriche reported fluorimetric determination of Al(III) with morin in an isobutyl methyl ketone–ethanol–water system. The Al–morin complex was measured at an emission wavelength of 495 nm with excitation at 418 nm under optimised conditions at pH 3.7–3.8. The method showed a linear range up to 40 μg L^−1^ and a reported detection limit of 0.2 μg L^−1^ [53]. The study also demonstrated interference from several ions, including Be^2+^, Zn^2+^, Pb^2+^, Fe^3+^, Cu^2+^, and F^−^, emphasising that the high sensitivity of fluorescence-based complexation does not eliminate the need for careful interference control [53]. Together with the immobilised-morin study [52], these results demonstrate the analytical potential of morin-based fluorescence while also illustrating the importance of chemical matrix conditions.

Computational chemistry can provide complementary information for understanding the molecular factors that control reagent behaviour. DFT calculations are particularly useful for examining molecular conformations, protonation sites, intermolecular interactions, and solvent effects. Safi and Wazzan investigated the azo dye PDPA using DFT calculations combined with pKa profiling, focusing on protonation sites, solvent effects, and molecular interactions with water [54]. Although this study was not performed on an immobilised SSA platform, it demonstrates how computational and experimental acid–base information can be combined to characterise the molecular states of an analytical azo compound. Such information may be useful during reagent selection and mechanistic interpretation, but calculated molecular properties should not be assumed to reproduce the behaviour of the reagent after immobilisation on a polymeric or fibrous support.

An additional example of polymer-supported optical detection is provided by PAR-functionalised thermosensitive ionic microgels. Zhou et al. synthesised 4-(2-pyridylazo)resorcinol-functionalised microgels and demonstrated optical detection of Cu^2+^, Mn^2+^, Pb^2+^, Zn^2+^, and Ni^2+^ in aqueous solution [55]. The reported colourimetric detection limits were 38 nM for Cu^2+^ at pH 3 and 12 nM at pH 7, while values of 14, 79, 20, and 21 nM were reported for Mn^2+^, Pb^2+^, Zn^2+^, and Ni^2+^, respectively, at pH 11 [55]. The optical response was attributed to chelation of the metal ions by the nitrogen atoms and ortho-hydroxyl groups of PAR within the microgel. Although this material is a thermosensitive ionic microgel rather than a fibrous PAN sorbent, it illustrates how immobilisation of a conventional chromogenic reagent within a polymeric microenvironment can produce nanomolar-level optical detection.

**Figure 3:**
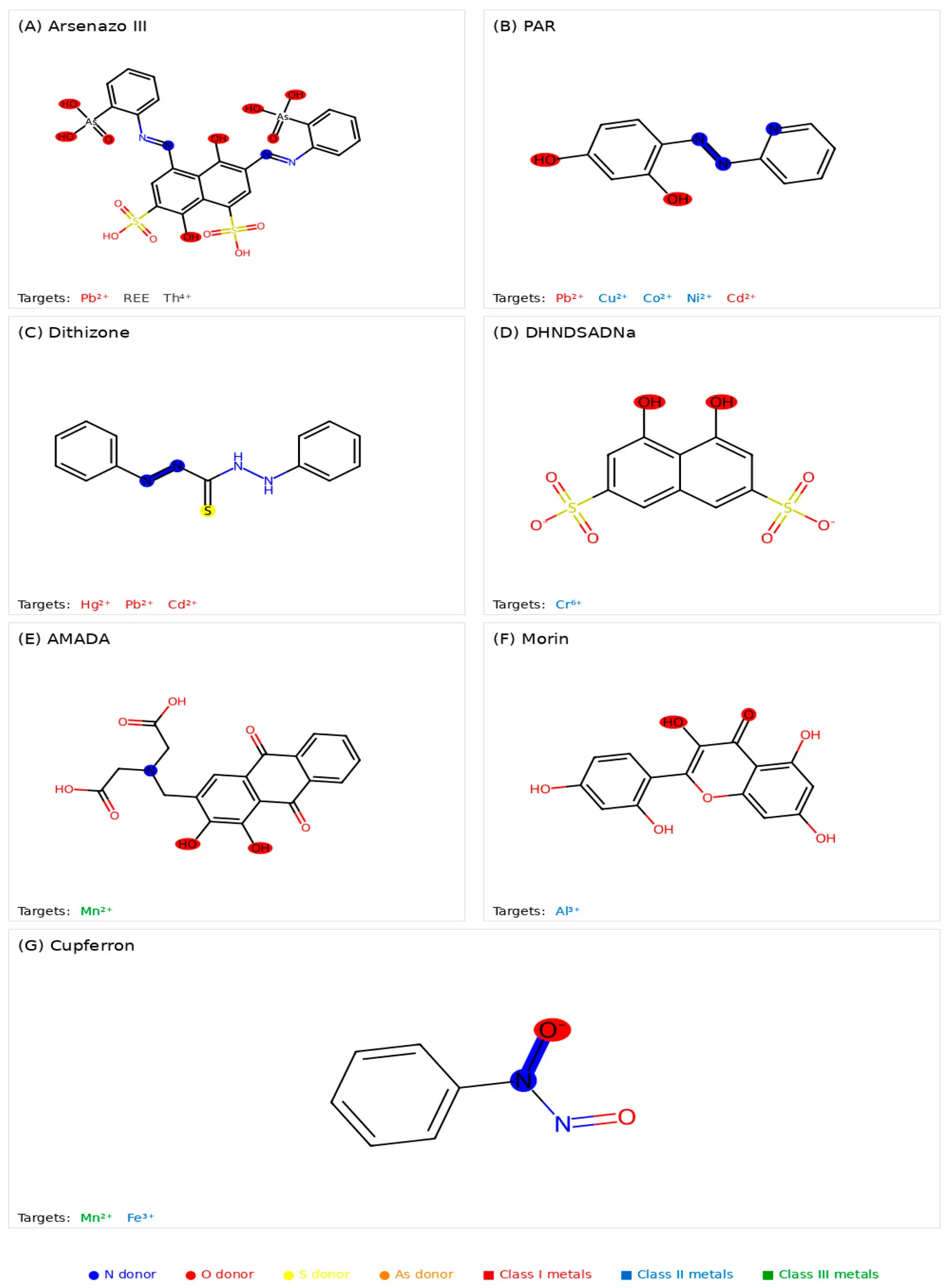
Structures of the principal immobilised reagents discussed in this review: (**A**) arsenazo III [PubChem CID 9570], (**B**) PAR [PubChem CID 71521], (**C**) dithizone [PubChem CID 2958], (**D**) DHNDSADNa [PubChem CID 5281072], (**E**) AMADA, (**F**) morin [PubChem CID 5281670], (**G**) cupferron [PubChem CID 10293]. Chelating donor atoms highlighted; target metal class indicated by symbol colour (red = Class I; blue = Class II; green = Class III). All structures freely available from PubChem (public domain, no permission required).

Taken together, these studies demonstrate two complementary directions for improving optical SSA. Immobilised fluorescent reagents such as morin can provide a fluorescence-based response for trace-metal sensing [52], while computational methods such as DFT and pKa profiling can help characterise the molecular states and interactions that influence reagent performance [53]. Polymer-supported chromogenic systems such as PAR-functionalised microgels further demonstrate that confinement of a ligand within a functional polymeric environment can yield nanomolar optical detection [54]. For fibrous PAN-based SSA, these approaches suggest useful design principles, but their performance should be evaluated experimentally because reagent speciation, immobilisation chemistry, accessibility of coordination sites, polymer microenvironment, and matrix interferences can substantially modify the analytical response. The chemical structures of the principal chromogenic and fluorescent reagents discussed in the following sections are summarised in Figure 3.

## 4. Analytical Performance of PAN-Based Fibrous Sorbents: Metal-Specific SSA

### 4.1. Lead, Pb(II)—Achieving Sub-WHO Sensitivity by Arsenazo III SSA

Lead is a priority contaminant in drinking water because of its toxicity and persistence in the environment. The WHO Guidelines for drinking-water quality give a provisional guideline value of 10 μg L^−1^ for lead, while Directive (EU) 2020/2184 establishes a parametric value of 5 μg L^−1^ to be met no later than 12 January 2036 [10,22]. These regulatory levels place stringent demands on analytical methods used for trace Pb(II) monitoring. The recently reported PPD-based sorption–spectrophotometric procedure using immobilised Arsenazo III provides a particularly sensitive PAN-based approach. Under optimised conditions (acetate buffer, pH 5.8; 10 min contact time; λmax = 640 nm), the method achieved a minimum detectable concentration (C_min_) of 0.24 μg L^−1^ and a relative standard deviation (Sr) of 0.06 [18]. The analytical response was linear over 0.04–2.4 μg mL^−1^ Pb(II), and the accuracy and reproducibility were evaluated using Student’s *t*-test and Fisher’s *F*-test; the reported values (t_exp_ = 1.09 < t_tab_ = 2.83 and Fexp = 2.52 < F_tab_ = 4.47) supported agreement with the comparative ICP-OES measurements [18]. The reported Cmin is therefore substantially below both the current WHO guideline value and the future EU parametric value, although regulatory compliance ultimately depends on the complete analytical procedure and quality-control requirements rather than on the detection limit alone [10,18,22].

The performance of the Arsenazo III/PPD system can be placed in context with other polymer-supported and solid-phase procedures for Pb(II). An Amido Black immobilised on a hexamethylenediamine-modified polyacrylonitrile fibre (PPD-1) was recently investigated using diffuse-reflectance measurements. The Pb(II) complex showed an analytical response around 490 nm, with optimum complex formation at pH 6–7 and an RSD of ≤2.9% [56]. Because the reported concentration range was 5–50 μg mL^−1^ and the publication does not provide the LOD unit consistently in its abstract, the numerical LOD should not be directly compared with the 0.24 μg L^−1^ Cmin reported for the Arsenazo III/PPD system [56]. This comparison nevertheless illustrates the broader use of chemically modified PAN fibres as supports for immobilised azo reagents.

A different PAN-based approach was developed using the PANV-AV-17 fibrous anion exchanger. Dedkova et al. demonstrated sequential determination of Hg(II), Cd(II), and Pb(II) from a single sample using three support disks and PAR or dithizone after sorption of anionic metal complexes [39]. Lead was determined after addition of iodide and sorption on the third disk, with a reported detection limit of 0.02 mg L^−1^ (20 μg L^−1^) for a 25 mL sample [39]. This method is analytically attractive because it permits multielement determination from a single aqueous sample, but its Pb detection limit is substantially higher than that of the Arsenazo III/PPD procedure [18,39].

Non-fibrous solid-phase and digital-image approaches provide additional benchmarks. A cellulose-based solid-phase extraction method combined with sodium rhodizonate and smartphone digital-image colourimetry was recently reported for Pb(II) determination in tap, river, and spring water. The method avoided a conventional elution step and provided recoveries of 93.5–97.5%, with relative standard deviations of 1.7–4.8%; several complementary greenness assessment tools indicated a favourable environmental profile [57]. Although such smartphone-assisted methods offer advantages in portability and reduced instrumentation requirements, their analytical sensitivity should be compared with fibrous SSA on the basis of experimentally validated LODs under comparable conditions.

For comparison with an instrumental atomic method, Zn-doped MoO_3_ nanorods have been used as a dispersive solid-phase extraction sorbent prior to high-resolution continuum-source atomic absorption spectrometry (HR-CS-AAS). The reported Pb(II) method provided a detection limit of 3.1 μg L^−1^ and extraction recoveries of 90–104% [58]. Thus, the Arsenazo III/PPD procedure reported a lower concentration threshold than this HR-CS-AAS-based extraction method, although the two approaches rely on fundamentally different detection principles and should not be ranked solely by LOD.

The selectivity of Arsenazo III-based Pb(II) SSA is controlled by the coordination chemistry of the reagent and by competing metal ions. In the PPD–Arsenazo III study, the optimised pH of 5.8 provided the analytical conditions for Pb(II) complex formation, while the influence of accompanying ions was evaluated experimentally [18]. More generally, Arsenazo III is known to form complexes with a range of metal ions, including Pb(II), and its analytical selectivity therefore depends strongly on pH, masking chemistry, and the composition of the sample matrix [43,44]. Consequently, claims concerning specific interferents and masking agents should be restricted to those experimentally demonstrated for the immobilised Pb(II) system rather than extrapolated from solution-phase Arsenazo III chemistry.

Overall, the available evidence identifies immobilised Arsenazo III on the PPD matrix as one of the most sensitive PAN-based SSA approaches currently reported for Pb(II), with a C_min_ of 0.24 μg L^−1^ under the stated experimental conditions [18]. Its principal advantages are the low concentration threshold, direct measurement on the polymeric support, and compatibility with simple spectrophotometric detection. At the same time, comparisons with other methods should distinguish between C_min_, LOD, LOQ, and method-specific detection criteria, because these parameters are not interchangeable across studies.

### 4.2. Mercury, Hg(II), and Methylmercury—Sensitivity Benchmarks for SSA and Magnetic SPE

Mercury is a priority toxic element requiring sensitive analytical control in environmental and food matrices. The WHO guideline value for inorganic mercury in drinking water is 6 μg L^−1^, while EFSA established a tolerable weekly intake (TWI) of 1.3 μg kg^−1^ body weight for methylmercury, reflecting particular concern regarding exposure through fish and other seafood [59]. These requirements have stimulated the development of preconcentration procedures that combine selective sorption with sensitive instrumental or spectrophotometric detection.

Dithizone remains an effective sulfur-containing chromogenic reagent for Hg(II) because of the strong affinity of its sulfur donor atoms for the soft Hg(II) ion. A representative solid-phase colourimetric approach was reported by Takahashi et al. [47], who prepared dithizone nanofibre-coated membranes for filtration-enrichment and direct colourimetric determination of Hg(II). More than 90% of 10 μg L^−1^ Hg(II) was retained on the dithizone nanofibre layer at pH 2.7, demonstrating efficient enrichment at the low-μg L^−1^ level. The method also showed tolerance toward several potentially interfering ions when EDTA was used as a masking reagent. Importantly, this study should be regarded as a ppb-level solid-phase colourimetric benchmark rather than evidence for sub-ng L^−1^ detection.

For comparison, considerably lower detection limits can be obtained when sorption is coupled to highly sensitive instrumental detection. Vicente-Martínez et al. [60] developed a magnetic solid-phase extraction procedure based on Fe_3_O_4_@GO and dithizone for the separation and speciation of Hg(II) and methylmercury, followed by ETAAS determination. Such magnetic SPE approaches demonstrate the analytical potential of selective sulfur-containing sorbents for mercury speciation; however, they should be distinguished from classical SSA because the analytical signal is generated by atomic spectrometry rather than by direct optical measurement of the sorbent phase.

A further sensitivity benchmark was reported by Li et al. [61] using magnetic poly(trithiocyanuric acid) polymers as an MSPE sorbent followed by ICP-MS detection. The optimised method provided an LOD of 0.61 ng L^−1^ for Hg(II), with a linear range of 2–3000 ng L^−1^ and a preconcentration factor of 232. The authors also reported rapid extraction, high adsorption capacity, and successful application to tap water, wastewater, lake water, and river water. This result illustrates the sensitivity attainable by combining sulfur-rich magnetic polymers with ICP-MS, but again represents an MSPE–ICP-MS procedure rather than an SSA method.

Within the specific framework of fibrous PAN-based SSA, the most directly relevant mercury procedure is the sequential method of Dedkova, Shvoeva, and Savvin [39] using PANV-AV-17. Hg(II), Cd(II), and Pb(II) were determined from a single 25 mL sample using three support discs. Mercury was first determined with dithizone after sorption from 0.2 mol L^−1^ NaCl solution, whereas Cd(II) was subsequently determined with PAR on the second disc. After addition of KI, Pb(II) was determined with PAR on the third disc. The reported detection limits were 0.01 mg L^−1^ for Hg(II) and 0.02 mg L^−1^ for Cd(II) and Pb(II). The procedure was validated using natural sodium chloride water, and the complete three-element determination required no more than approximately 50 min for five or six samples.

Thus, the available evidence indicates a clear distinction between two analytical levels. Classical PAN-fibre SSA provides simple, reagent-efficient, and potentially field-compatible solid-phase optical determination, exemplified by the PANV-AV-17 sequential procedure, whereas modern magnetic SPE coupled with ETAAS or ICP-MS can achieve sub-ng L^−1^ detection. The latter approaches are therefore best considered sensitivity benchmarks rather than direct replacements for SSA. For PAN-based fibrous sorbents, the principal analytical advantage remains the integration of selective sorption and optical detection on a compact solid support, with opportunities for further improvement through optimised reagent immobilisation, sorbent morphology, and preconcentration design.

### 4.3. Copper(II), Cu(II)—Selectivity, pH Dependence, and Emerging Solid-Phase Optical Methods

Copper is an essential trace element, but excessive exposure can produce adverse health effects. The current WHO drinking-water guideline value for copper is 2 mg L^−1^, while the adult recommended dietary allowance is 0.9 mg day^−1^ [10,62]. For analytical monitoring, the principal challenge is therefore not simply copper detection, but selective determination in the presence of other transition and alkaline-earth-metal ions and over a broad concentration range.

Among PAN-based solid-phase optical procedures, recent work by Ruzmetov et al. [30] is particularly relevant to the present review. The authors developed polyacrylonitrile-derived fibrous sorbents PPF-1 and PPA-1 containing immobilised Thoron I for Cu(II) sorption from technogenic, waste, and industrial waters. The immobilised reagent produced a distinct solid-phase spectral response: binding of Cu(II) shifted the diffuse-reflectance band of Thoron I toward approximately 490 nm and changed the fibre colour from violet to orange–brown. Importantly, the Cu(II) extraction increased with pH and reached a maximum at approximately pH 6–8, illustrating the central role of protonation and competing hydrolysis processes in defining the working selectivity window. The method was based on sorption coupled with atomic-absorption determination, while diffuse-reflectance measurements were additionally used to characterise the solid-phase optical response; therefore, it should not be presented as a pure SSA procedure with a spectrophotometric LOD [30].

Earlier PAN-fibre studies provide direct evidence that copper can also be determined by solid-phase diffuse-reflectance measurements. Dedkova et al. [29] investigated Cu(II) adsorption on PAN fibre containing the KU-2 cation exchanger and immobilised 1-(2-pyridylazo)-2-naphthol (PANF-KU-2-PAN). After adsorption from a 20-mL sample containing 0.01 mol L^−1^ HCl, the copper complex was measured at 640 nm by diffuse-reflectance spectroscopy or visually using a colour scale. The procedure covered 0.05–0.40 μg mL^−1^ Cu(II), with an LOD of 0.03 μg mL^−1^ and an analysis time of approximately 30–35 min for five to six samples. At 0.1 μg mL^−1^ Cu(II), equal concentrations of Co, Zn, and Pb and twofold concentrations of Ag, Fe(III), Cd, Mn(II), Bi(III), and Cr(III) did not interfere, demonstrating the practical selectivity achievable with the PANF–KU-2–PAN platform [29].

Multielement PAN-based sorption–spectroscopic analysis was demonstrated by Shvoeva et al. [63], who developed a two-layer PAN adsorbent containing PANV-AV-17 and PANV-KU-2. In flow-through operation, V(V) and Cr(VI) were concentrated on the anion-exchange layer, whereas Ni(II) and Cu(II) were retained on the cation-exchange layer. Copper was subsequently determined on the PANV-KU-2 disk with sodium diethyldithiocarbamate after removal of the nickel complex. The applicable concentration range for Cu(II) was 0.02–0.15 μg mL^−1^ in simultaneous determinations with V, Cr, and Ni, with reported RSD values below 20%. This study illustrates how spatially separated sorption sites can reduce inter-element competition without requiring complete chemical separation of the sample before the optical measurement [63].

A different strategy was reported by Guo et al. [64], who coupled cation-exchange fibre SPE with UV–Vis spectrophotometry and partial least-squares regression for simultaneous determination of Cu(II), Co(II), and Ni(II). The fibre-based preconcentration step provided enrichment greater than 80-fold, while chemometric treatment resolved strongly overlapping absorption spectra. LODs were 0.10 μg L^−1^ for Cu(II), 0.15 μg L^−1^ for Co(II), and 0.13 μg L^−1^ for Ni(II), with RSD values below 5%. Because the metals were measured after elution from the fibre, this is an SPE–UV–Vis/PLS method rather than direct SSA; nevertheless, it provides an important benchmark showing how fibrous preconcentration combined with multivariate calibration can substantially improve Cu(II) sensitivity and simultaneous analysis [64].

Recent non-PAN sorbents further demonstrate the rapid development of selective solid-phase Cu(II) preconcentration. Turan et al. [65] used an activated-carbon-based Cu(II)-ion-imprinted sorbent for SPE–FAAS. The ion-imprinted material showed a maximum Langmuir adsorption capacity of 312.5 mg g^−1^ and provided an LOD of 0.038 μg L^−1^, with successful validation using tap water and certified reference materials. Although the analytical readout was FAAS rather than solid-phase optical detection, this work illustrates the performance advantage that highly selective recognition sites can provide when Cu(II) preconcentration is coupled to a sensitive instrumental detector [65].

The trend toward portable and environmentally benign solid-phase sensors is illustrated by Minabi-Nezhad et al. [66], who incorporated anthocyanins extracted from *Syzygium cumini* into bacterial-cellulose nanofibres. The resulting BCNF material exhibited a visible response to Cu(II) and showed selectivity over Pb(II), Co(II), Cd(II), Ni(II), Hg(II), Mn(II), and other tested ions. The reported detection limits were 10–400 mg L^−1^ for liquid samples and 50–500 mg kg^−1^ for solid samples, placing this system well above the sensitivity of instrumental SPE methods but demonstrating the potential of renewable fibrous supports for inexpensive visual screening [66].

More recently, Zhang et al. [67] reported a portable bacterial-cellulose fluorescent film containing yellow-emitting carbon dots for Cu(II) detection in food and environmental samples. The bacterial-cellulose matrix improved sensor stability and portability, while Cu(II)-dependent fluorescence quenching enabled rapid optical detection. Smartphone-assisted analysis provided a practical route toward visual interpretation within approximately 1 min. This system represents an important evolution from conventional bulk spectrophotometry toward portable fibrous optical sensors, although it is a fluorescent sensor rather than classical sorption–spectroscopic analysis of an immobilised chromogenic reagent [67].

Taken together, these studies show that Cu(II) selectivity in fibrous analytical systems is strongly controlled by the chemical environment of the immobilised reagent, the ion-exchange functionality of the carrier, and the pH-dependent competition between protonation, complex formation, and hydrolysis. PAN-based systems remain particularly attractive because the fibre can simultaneously provide mechanical support, rapid mass transfer, and a defined optical response. The most promising direction is therefore not simply lowering the LOD, but integrating selective immobilised reagents, controlled pH domains, multielement recognition, and direct or portable optical readout into a single fibrous platform.

### 4.4. Cadmium, Cd(II)—HSAB Selectivity Without Masking

Cadmium is a toxic and carcinogenic trace element for which the kidney is the principal target organ during chronic exposure. The WHO guideline value for cadmium in drinking water is 3 μg L^−1^, while the Joint FAO/WHO Expert Committee on Food Additives established a provisional tolerable monthly intake of 25 μg kg^−1^ body weight [10]. WHO notes that cadmium has a long biological persistence and that renal tubular dysfunction is the critical health endpoint considered in the derivation of the current guideline value. These characteristics make sensitive preconcentration particularly important for trace Cd(II) determination in environmental and food matrices.

Dithizone remains one of the most established chromogenic ligands for Cd(II) because of its ability to form coloured metal complexes through sulfur- and nitrogen-donor atoms. However, the analytical selectivity of dithizone-based systems is governed by more than the HSAB classification of Cd(II): pH, ligand protonation, competing ions, reagent loading, and the physicochemical properties of the solid support all influence extraction efficiency. Consequently, experimentally established operating pH ranges and interference studies are more informative for analytical evaluation than numerical Pearson softness parameters alone.

A particularly relevant precedent for solid-phase spectrophotometry is the micro-solid-phase spectrophotometric method of Santos et al. [68]. In a sequential-injection lab-on-valve platform, nitrilotriacetic-acid (NTA) beads were packed into a miniaturised flow cell and used to retain Cd(II), Zn(II), and Cu(II). After complex formation with dithizone, the coloured products were measured directly on the surface of the NTA resin at 550 nm. The method gave preconcentration factors of 11–21 and detection limits of 0.23 μg L^−1^ for Cd(II), 2.39 μg L^−1^ for Zn(II), and 0.11 μg L^−1^ for Cu(II), demonstrating that direct solid-phase optical measurement can reach the low-μg L^−1^ range while avoiding a conventional elution step. The successful application to freshwater samples also demonstrates the analytical relevance of this approach for real matrices.

A more recent solid-phase optical strategy was reported by Mahmoudian et al. [69] using thin-film microextraction with a porous polypropylene membrane modified with n-octanol and dithizone. Cd(II) was extracted directly onto the membrane, and the resulting Cd–dithizone complex was quantified by smartphone-based RGB colourimetry. The method provided a linear range of 0.5–300 μg L^−1^ and an LOD of 0.1 μg L^−1^, with recoveries of 95.0–103.0% and RSD values below 2.5% for spiked water and food samples. Although the carrier was polypropylene rather than PAN, this work is highly relevant to PAN-based SSA because it combines immobilised chromogenic chemistry, solid-phase enrichment, and direct optical readout without requiring elution before measurement.

The trend toward portable and low-cost solid-phase colourimetry is further illustrated by Litluechai et al. [70], who developed a Cd(II) colourimetric system based on silica sol modified with dithizone and dodecyltrimethylammonium bromide. Cd(II) complexation with dithizone produced a pronounced colour change from purple to orange, enabling UV–Vis as well as customised image-based colour analysis. Under optimised conditions, the method showed a linear range of 0.01–0.25 mg L^−1^ and an LOD of 5.0 μg L^−1^. Although its sensitivity was lower than that of the thin-film microextraction approach, the system demonstrates the continuing movement toward simple, portable colourimetric determination without sophisticated instrumentation.

For highly selective preconcentration, ion-imprinted sorbents offer an alternative to non-specific chelating phases. Islam and Rais [71] developed a magnetic Fe_3_O_4_@MWCNT ion-imprinted polymer for Cd(II) separation and preconcentration before FAAS determination. The sorbent exhibited a maximum adsorption capacity of 109 mg g^−1^, approximately 2.5 times that of the corresponding non-imprinted polymer. Under optimised conditions, the SPE–FAAS procedure provided an LOD of 1.13 μg L^−1^ with a preconcentration factor of 50 and was successfully applied to food, water, and wastewater samples. These results emphasise that selective recognition sites can substantially improve matrix tolerance, although the approach remains instrumental SPE rather than direct SSA.

A sulfur-rich polymeric sorbent provides another recent benchmark. Altunay et al. [72] developed a polyvinyl benzyl xanthate adsorbent containing pendant sulfide groups for vortex-assisted dispersive micro-solid-phase extraction of Cd(II), followed by FAAS. The optimised method covered 0.20–150 μg L^−1^, with an LOD of 0.06 μg L^−1^, LOQ of 0.20 μg L^−1^, RSD of 4.3%, and a preconcentration factor of 160. The material was applied to both food and water samples. This study is particularly useful for comparison with dithizone-based PAN systems because it demonstrates how accessible sulfur donor groups on a polymeric support can improve Cd(II) preconcentration without requiring a conventional packed-column format.

The most recent fibrous example is provided by Carrizo et al. [73], who introduced thiocarbanilide-functionalised carbon nanofibres for dispersive micro-solid-phase extraction of Cd(II) in bottled cola soft drinks followed by hydride-generation atomic fluorescence spectrometry. The study demonstrates the growing use of sulfur-containing functionalised nanofibres as high-surface-area sorbents for selective Cd(II) enrichment in complex food matrices. Because the analytical signal was ultimately obtained by atomic fluorescence spectrometry, the method should be regarded as a modern sorption benchmark rather than as SSA. Nevertheless, the material design is highly relevant to future PAN-fibre platforms, particularly with respect to surface accessibility, sulfur-rich recognition sites, and rapid dispersive extraction.

Taken together, the available evidence shows that Cd(II) selectivity in solid-phase analytical systems is controlled by the combined effects of ligand donor atoms, pH, sorbent morphology, and competing-ion chemistry rather than by HSAB classification alone. Direct solid-phase spectrophotometry has already demonstrated Cd(II) detection in the sub-μg L^−1^ range, while modern membrane colourimetry and sulfur-rich fibrous SPE provide complementary routes toward improved sensitivity and portability. For PAN-based SSA, the most credible development strategy is therefore the integration of immobilised chromogenic ligands, optimised pH domains, high-accessibility fibre morphologies, and direct optical readout. Such a combination could improve selectivity and preconcentration while avoiding the additional dilution and procedural complexity associated with elution-based SPE.

### 4.5. Chromium—Ratiometric Speciation of Cr(III) and Cr(VI) Without Chromatography

Chromium speciation is analytically important because the environmental behaviour and toxicity of chromium depend strongly on its oxidation state. Cr(VI) is substantially more toxic and is classified as carcinogenic to humans by IARC, whereas Cr(III) is considerably less toxic and is involved in essential biochemical processes. The WHO drinking-water guideline value is currently 50 μg L^−1^ for total chromium. Importantly, WHO retains a total-chromium guideline because reliable routine speciation in drinking water remains analytically challenging [10].

Solid-phase spectrophotometry provides an attractive alternative to chromatographic separation because one oxidation state can be selectively retained or chemically converted before direct measurement of the enriched solid phase. A representative method was reported by Amin and Kassem [74], who developed a solid-phase spectrophotometric procedure based on the formation of a Cr(III) complex with 4-(2-benzothiazolylazo)-2,2′-biphenyldiol (BTABD) and sorption onto a dextran-type anion-exchange gel (Sephadex DEAE A-25). Cr(VI) was determined by difference after reduction of the total chromium fraction. The absorbance of the loaded gel was measured directly at 628 and 750 nm. Using 35 mg of exchanger, detection limits of 13 ng L^−1^ for a 500 mL sample and 8 ng L^−1^ for a 1000 mL sample were reported, with calibration over 0.05–1.45 μg L^−1^ and RSD below 1.85%. This study demonstrates that direct solid-phase optical measurement can provide very high sensitivity for chromium speciation when large sample volumes are coupled with selective preconcentration.

A more directly relevant PAN-based example was reported by Bobojonov et al. [41], who immobilised 4,5-dihydroxynaphthalene-2,7-disulfonic acid disodium salt dihydrate (DHNDSADNa) on a polyethylene-polyamine polyacrylonitrile (PPA) matrix for Cr(VI) determination. The immobilised reagent produced distinct spectral responses near 460 and 590 nm for the reagent and its Cr(VI) reaction product, respectively. A detection limit of 2 μg mL^−1^ was reported, together with high selectivity in the presence of Co(II), Ni(II), Al(III), Fe(II), Ti(IV), Zr(IV), Ta(V), Mn(II), and W(VI). The study therefore provides valuable evidence for selective immobilised-reagent chemistry on a PAN-derived fibrous carrier. However, it should be described as a Cr(VI)-selective solid-phase method rather than as a complete chromatography-free ratiometric determination of both Cr(III) and Cr(VI) [41].

The relatively high LOD of the DHNDSADNa/PPA system also defines its practical application range. At 2 mg L^−1^, the reported detection limit is approximately 40 times higher than the WHO guideline value of 50 μg L^−1^ for total chromium. Accordingly, this particular PAN-based procedure is better regarded as a method for relatively concentrated contaminated or industrial waters than as a drinking-water compliance method. Such a limitation is important when comparing immobilied-reagent SSA with highly sensitive instrumental preconcentration procedures.

Recent magnetic solid-phase extraction approaches provide a substantially different sensitivity benchmark. Morales-Benítez et al. [75] developed a magnetic sorbent based on magnetic nanoparticles coupled to graphene oxide functionalised with 4-aminobenzenesulfonic acid (M@GO-ABS) and integrated the extraction online with ICP-OES. The system was designed for Cr(III)/Cr(VI) speciation in environmental waters and was validated for samples including saline matrices such as seawater. This approach demonstrates the advantage of magnetic preconcentration and automated instrumental detection for chromium speciation, while remaining fundamentally different from direct SSA because the analytical signal is obtained by ICP-OES rather than from the loaded solid phase.

A recent optical alternative was reported by Darwish et al. [76] using a cellulose triacetate membrane optode containing 1,3-benzenediamine,N,N′-bis(2-furanylmethylene) (BDBFM), with Aliquat 336 incorporated to facilitate immobilisation of the chromophore and the Cr(VI)–BDBFM complex. The membrane develops a concentration-dependent violet colour in response to Cr(VI), enabling optical determination without chromatographic separation. Importantly, the same optode platform was extended to chromium speciation: Cr(VI) can be determined directly, while total chromium is obtained after oxidation of Cr(III) to Cr(VI), allowing Cr(III) to be calculated by difference. This approach is particularly relevant to the development of portable solid-phase optical sensors, although it uses a cellulose-triacetate membrane rather than PAN.

Another recent development is the use of automated spectrophotometric analysis with 1,5-diphenylcarbazide (DPC). Dikobe et al. [77] reported a sequential discrete robotic analyser in which Cr(VI) reacts with DPC and Cr(III) is obtained by difference after online oxidation of Cr(III) to Cr(VI). The method gave detection limits of 0.004 mg L^−1^ for Cr(VI) and 0.015 mg L^−1^ for total chromium, with RSD values below 2% and generally satisfactory recoveries of 90–110% in environmental samples. Although this system does not employ a fibrous sorbent, it is a useful current benchmark for rapid non-chromatographic chromium speciation by optical means.

Taken together, these studies show three distinct analytical strategies for Cr(III)/Cr(VI) speciation. Classical solid-phase spectrophotometry can provide extremely low detection limits when large sample volumes are preconcentrated on ion-exchange media [74]. PAN-derived immobilised reagents such as DHNDSADNa/PPA offer a more compact solid-phase format with strong Cr(VI) selectivity, but the demonstrated detection limit is currently more appropriate for contaminated waters. Modern magnetic SPE–ICP-OES and membrane-optode approaches provide broader opportunities for automated or portable speciation, while avoiding chromatographic separation [76,77]. For PAN-based SSA, the main opportunity is therefore to combine the selective chemistry of immobilised reagents with higher-capacity fibrous preconcentration and direct optical readout, with particular attention to independently validated Cr(III)/Cr(VI) speciation rather than inferring one oxidation state solely from total chromium.

### 4.6. Manganese, Mn(II)—Complementary pH Windows of Cupferron and AMADA

Manganese is an essential trace element, but excessive exposure, particularly chronic inhalation or ingestion, can produce neurological effects including manganism. For drinking water, the World Health Organization currently specifies a provisional guideline value of 0.08 mg L^−1^ (80 μg L^−1^) for total manganese, derived primarily to protect sensitive populations against potential neurological effects [10]. Manganese concentrations in groundwater can substantially exceed this value, particularly under reducing conditions and in association with iron-rich aquifers, making selective and sufficiently sensitive determination important for environmental monitoring. WHO also notes that manganese concentrations in fresh waters typically range from approximately 1 to 200 μg L^−1^, although much higher concentrations can occur in groundwater and polluted waters [10].

Cupferron provides a useful example of a nitroso reagent immobilised on a PAN-derived fibre for direct solid-phase optical determination. Smanova et al. [51] recently reported Mn(II) determination using cupferron immobilised on a PPD fibrous polyacrylonitrile sorbent. The analytical response was measured by diffuse-reflectance spectroscopy, with the Mn-containing system monitored at approximately 500 nm. The optimum detection medium was strongly acidic, with pH 3 identified as the working condition; the authors also observed that precipitation occurred under neutral and alkaline conditions. Under the reported conditions, the procedure showed a linear response over 0.5–5.0 mg L^−1^ and was proposed for determination of Mn(II) in industrial wastewater. The study is therefore directly relevant to PAN-based SSA, but its demonstrated concentration range places it primarily in the field of contaminated and industrial waters rather than trace-level drinking-water compliance.

The strongly acidic working range of the cupferron system is analytically important. Rather than attributing the selectivity solely to differences in complex stability with Fe(III), it is more appropriate to describe the method in terms of the experimentally established pH window. At pH ≈ 3, the PAN–cupferron system generates an optical response for Mn(II), whereas neutral and alkaline conditions lead to loss of the desired analytical performance, including precipitation. Thus, the principal advantage of the method is the combination of immobilised reagent chemistry and direct diffuse-reflectance measurement rather than an experimentally demonstrated universal suppression of iron interference [51].

A second PAN-based strategy uses the immobilised ligand *AMADA*. Madatov et al. [42] developed alizarin-3-methylamino-N,N-diacetic acid (AMADA) immobilised on a polyethylene-polyamine-modified polyacrylonitrile matrix (AMADA/PPP) for Mn(II) determination. The immobilised reagent and its Mn(II) complex exhibited characteristic bands at approximately 500 and 580 nm, respectively. The reported determination limit was 0.02 μg mL^−1^ (20 μg L^−1^), placing the method in a concentration range relevant to environmental waters around the WHO provisional guideline value. The authors also reported high selectivity in the presence of Ca(II), Mg(II), Cd(II), Zn(II), and Fe(II), together with good repeatability and the possibility of regeneration of the modified material. Importantly, the selectivity should be described empirically rather than attributed to a specific rigid five-membered catecholate geometry, because the published evidence establishes interference resistance but does not justify such a mechanistic simplification [42].

The difference between these two PAN-based reagent systems is therefore most clearly expressed through their experimentally established operating domains. The cupferron/PPD system is operated under strongly acidic conditions (approximately pH 3) and is demonstrated for relatively concentrated industrial waters, whereas AMADA/PPP provides Mn(II) determination at substantially lower concentrations and shows tolerance toward several common competing metal ions. This complementarity is analytically useful because the optimal reagent/support combination depends on both matrix composition and expected Mn concentration rather than on a single universally optimal pH.

Recent non-PAN optical methods provide useful benchmarks for the sensitivity and portability of Mn(II) detection. Panjina et al. [78] developed a smartphone-based colourimetric method using silver nanoparticles synthesised with okara extract and citrate. Mn(II) was detected over 0.025–5.0 mg L^−1^, with an LOD of 0.01 mg L^−1^ (10 μg L^−1^), without pH adjustment and with a reaction time of approximately 9 min. The method was validated using filtered water samples against atomic-absorption spectrometry. Although no solid PAN support was used, this work demonstrates the movement toward low-cost digital colourimetry for on-site Mn monitoring.

A related smartphone-assisted procedure based on the periodate oxidation of 3,3′,5,5′-tetramethylbenzidine achieved an Mn(II) LOD of 0.2 μg mL^−1^ (200 μg L^−1^) over a working range of 0.1–2.5 μg mL^−1^. The method was demonstrated for freshwater samples and showed good agreement with ICP-OES results [79]. Although its sensitivity is lower than that of the *AMADA*/PPP system, it illustrates the growing importance of portable image-based analysis as a complementary strategy to conventional spectrophotometry.

A further recent development is the 2025 PPD–cupferron study of Smanova et al. [51], which confirms that immobilised nitroso reagents can retain their analytical functionality on PAN-based fibrous supports and generate measurable diffuse-reflectance responses. Together with *AMADA*/PPP, these studies demonstrate that PAN fibres can accommodate chemically different ligands while maintaining direct optical transduction [42,51].

Overall, the evidence supports a complementary rather than universal approach to Mn(II) determination. Cupferron/PPD offers a simple acidic solid-phase optical procedure suited to relatively concentrated industrial samples, whereas AMADA/PPP extends PAN-based analysis toward lower concentrations and demonstrates substantial tolerance to common matrix ions. Modern smartphone-based colourimetry further shows that portable detection is becoming increasingly practical. For PAN-based SSA, future development should therefore focus on lowering the working concentration range of immobilised-reagent systems while preserving direct solid-phase optical readout, chemical stability, and experimentally demonstrated resistance to Fe and other competing ions.

### 4.7. Nickel, Cobalt, and Zinc—Multi-Element Approaches and Chemometric Resolution

Ni(II), Co(II), and Cu(II) can occur simultaneously in environmental and industrial water samples, creating analytical difficulties because their absorption spectra may substantially overlap. A particularly relevant solid-phase spectrophotometric approach combines solid-phase extraction with partial least-squares (PLS) regression to resolve the overlapping UV–Vis responses of these ions. In the reported procedure, the metal ions were preconcentrated on cation-exchange fibres, and the enriched phase was subsequently analysed by UV–Vis spectrophotometry with PLS calibration. The analytes were enriched by more than 80-fold, while the limits of detection were 0.10 μg L^−1^ for Cu(II), 0.15 μg L^−1^ for Co(II), and 0.13 μg L^−1^ for Ni(II), with relative standard deviations below 5%. The study demonstrates the practical value of chemometric resolution when strongly overlapping spectral responses prevent reliable simultaneous determination by conventional univariate calibration [64].

For individual Co(II) determination, solid-phase preconcentration using PAR-loaded Amberlite XAD-7 has also provided high sensitivity. In mineral-water samples, cobalt was retained on the PAR-loaded resin at pH 2.0 ± 0.2 and subsequently determined by flame atomic absorption spectrometry. The reported detection limit was 0.1 ng mL^−1^ (0.10 μg L^−1^), with recoveries of up to 90%. The method was applied to natural mineral waters containing Co at concentrations of 0.5–3.5 ng mL^−1^ [80].

For Ni(II), activated electrospun PAN nanofibres (PANmod) have demonstrated preferential adsorption of Ni(II) relative to Pb(II). The PAN nanofibres were modified with NaHCO_3_, and the resulting material showed increasing adsorption capacity with pH, reaching a maximum at pH 7.0. Equilibrium was attained within approximately 60 min, and the adsorption kinetics were described by a pseudo-second-order model. The authors reported higher affinity of PANmod for Ni(II) than for Pb(II), demonstrating that chemical modification of the PAN surface can substantially alter its metal–ion retention behaviour [35].

For Zn(II), 2,3-dihydroxypyridine (DHP)-loaded cellulose has been investigated as a macromolecular solid extractant. The material exhibited substantial sorption capacity toward Zn(II) and other divalent metal ions, with quantitative sorption of Zn(II) reported in the pH range of 6.0–8.0. The study further demonstrated that simultaneous sorption of several metal ions was possible at pH 7.0 when the total metal concentration did not exceed the sorption capacity of the material [81].

Taken together, these studies demonstrate three complementary strategies for Ni(II), Co(II), and Zn(II): chemometric resolution of overlapping spectra for simultaneous multi-element determination, selective solid-phase preconcentration for individual trace-metal measurements, and functionalisation of polymeric or cellulose supports to modify metal–ion retention. The analytical performance depends not only on the intrinsic metal–ligand affinity but also on the sorbent chemistry, pH, preconcentration procedure, sample matrix, and instrumental detection mode. Accordingly, LOD values obtained with different sorbents and detection techniques should be compared cautiously rather than interpreted solely in terms of metal–ligand hardness or softness. The metal-specific analytical figures of merit discussed above are consolidated in Table 4.

## 5. Applications in Real Sample Matrices—Validation Strategy and Analytical Performance

### 5.1. Environmental and Industrial Waters—Matrix Complexity and Interfering Species

Natural surface waters are chemically heterogeneous matrices in which dissolved organic matter (DOM), suspended particles, major inorganic ions, and coexisting trace metals can influence both analyte retention and the optical response of solid-phase analytical systems. Humic and fulvic substances are important components of DOM and contain functional groups capable of binding metal ions; consequently, DOM can modify metal speciation, mobility, and availability for interaction with an analytical sorbent. The extent of this effect depends strongly on pH, ionic composition, the nature of the metal ion, and the properties of the sorbent. Therefore, matrix effects observed for conventional sorbents or solution-phase systems should not be quantitatively transferred to PAN-based fibrous supports without experimental validation [82,83].

For PAN-based sorption–spectroscopic analysis (SSA), matrix effects are particularly relevant because metal–ion retention and optical response occur at or within a chemically functionalised polymer phase. The PPD–Arsenazo III system for Pb(II), based on Arsenazo III immobilised on a polyacrylonitrile/polyethylenepolyamine/1,2-dichloroethane matrix, provides a representative example. The procedure operates at pH 5.8 with a maximum analytical response at 640 nm and a reported minimum detectable concentration of 0.24 μg L^−1^. The authors also demonstrated a linear response over 0.04–2.4 μg mL^−1^ and applied the method to water samples. Statistical evaluation using Student’s *t*-test and Fisher’s *F*-test supported the accuracy and reproducibility of the proposed procedure [18].

The AMADA/PPP system provides a second example of PAN-supported analysis in which the effects of coexisting ions were explicitly considered. Alizarin-3-methylamino-N,N-diacetic acid (AMADA) was immobilised on a polyethylene-polyamine-modified PAN matrix for Mn(II) determination. The system exhibited characteristic analytical bands at approximately 500 and 580 nm, and the reported determination limit was 0.02 μg mL^−1^. Selectivity studies included Ca(II), Mg(II), Cd(II), Zn(II), and Fe(II), demonstrating that the analytical response depended on the composition of the accompanying matrix rather than on Mn(II) concentration alone [42]. Such interference studies are essential when transferring immobilised-reagent procedures from simple laboratory standards to industrial or environmental waters.

A related example is provided by DHNDSADNa immobilised on a polyethylene-polyamine-modified PAN matrix for Cr(VI) determination. The immobilised reagent exhibited an absorption band near 460 nm, whereas the Cr(VI)-containing system showed a pronounced band near 590 nm. The reported detection limit was 2 μg mL^−1^. The study investigated the influence of several potentially interfering ions, including Co(II), Ni(II), Al(III), Fe(II), Ti(IV), Zr(IV), Ta(V), Mn(II), and W(VI), and demonstrated selective response to Cr(VI) under the specified experimental conditions [41]. This type of interference study is particularly relevant to industrial waters, where Cr(VI) may occur together with other transition and oxyanion-forming elements.

The available interference data allow a more specific distinction between cationic and anionic matrix effects. In covalently functionalised PAR–PAN, Ca(II) and Mg(II) produced no comparable colour change to the target heavy-metal ions [38], and the AMADA/PAN system was evaluated in the presence of Ca(II), Mg(II), Cd(II), Zn(II), and Fe(II) [42]. For the anion-exchange PAN-T platform, competitive-ion experiments showed retained Cr(VI) selectivity in the presence of common anions including Cl^−^, NO_3_^−^, and SO_4_^2−^ [28]. These results are useful but do not establish universal matrix tolerance for all PAN fibres.

Systematic PAN-SSA data for carbonate/bicarbonate, phosphate, and dissolved organic matter are much more limited. Carbonate and phosphate can change metal speciation and compete through complexation or surface interactions, while humic/fulvic components can bind metals and contribute their own absorbance or scattering background. Their effects should therefore be measured rather than assumed negligible. A minimum environmental validation set should report water hardness (Ca/Mg), alkalinity, chloride, sulfate, phosphate, representative competing transition metals, dissolved organic carbon, turbidity, and pH together with recovery or calibration-bias data.

Older PAN-based fibre studies also demonstrate the feasibility of analysing several toxic metals from a common water sample. Dedkova et al. developed a sequential procedure on PANV-AV-17 for separate determination of Hg(II), Cd(II), and Pb(II). The metals were retained as anionic complexes and subsequently determined using immobilised dithizone or PAR on separate fibre discs. The reported detection limits were 0.01 mg L^−1^ for Hg(II) and 0.02 mg L^−1^ for Cd(II) and Pb(II), using a 25-mL sample volume. The procedure was validated by the added–found method using natural sodium-chloride water, demonstrating the applicability of the fibrous sorbent to a real aqueous matrix [39].

Non-PAN solid-phase extraction methods provide useful external benchmarks for matrix tolerance, but they should be distinguished clearly from direct PAN-based SSA. For example, salicylic-acid-functionalised silica-coated magnetite nanoparticles have been used for preconcentration of Cu(II), Cd(II), Ni(II), and Cr(III) from environmental and other real samples. The study examined matrix-ion effects and applied the procedure to real samples after acid elution and instrumental determination. Because the final measurement was performed after elution rather than directly on the sorbent, this system is best considered a comparative SPE benchmark rather than a PAN-SSA method [84].

A more recent magnetic SPE–FAAS system based on hierarchical MnSb_2_O_6_@Fe_3_O_4_ nanoparticles provides another example of validation in complex matrices. The method was developed for Cu(II) and provided a reported detection limit of 2.1 ng mL^−1^, a 48-fold enhancement factor, and a linear range of 10–200 ng mL^−1^. Recoveries of 93–102% were reported for tap water, wastewater, and apple-tea samples [85]. Although these results demonstrate successful matrix tolerance in magnetic SPE, the analytical signal was obtained by FAAS after separation and therefore should not be interpreted as direct evidence for the performance of PAN-based SSA.

Real-sample validation should therefore be judged by matrix composition as well as nominal LOD. Standard addition or matrix-matched calibration is preferable when the sample contains substantial hardness, salinity, dissolved organic matter, or coexisting transition metals. Recovery should be tested at more than one concentration level and, where possible, compared with an established reference technique. The main evidence gap is not the absence of any successful water applications, but the lack of harmonised interference panels across different PAN architectures.

### 5.2. Soils and Sediments—Digestion Efficiency, Matrix Matching, and CRM Validation

Soils and sediments are substantially more complex analytical matrices than natural waters because trace metals can be distributed among exchangeable, carbonate-associated, Fe/Mn-oxide-associated, organic/sulfide-bound, and residual mineral fractions. Consequently, the reported metal concentration depends not only on the instrumental method but also on the operational definition of the analyte fraction and on the sample-preparation procedure used before preconcentration or measurement [86].

For microwave-assisted preparation of soils and sediments, U.S. EPA SW-846 Method 3051A is a commonly used approach. The method is designed as a microwave-assisted acid digestion/extraction procedure using concentrated HNO_3_, or HNO_3_ together with HCl, and is applicable to sediments, sludges, soils, and related solid matrices. Importantly, Method 3051A should not be interpreted as a universal procedure for complete dissolution of refractory silicate phases; the extent of extraction depends on the matrix and analyte. Therefore, results obtained after 3051A should be described according to the extraction procedure actually applied rather than automatically labelled as total elemental concentrations [86].

Aqua-regia extraction provides another widely used operational approach for soil trace-element determination. ISO 11466:1995 [87] specifies extraction of trace elements soluble in aqua regia from soils and related materials containing less than approximately 20% organic carbon. However, this standard has been withdrawn, and it should therefore be cited as a historical or established reference procedure rather than as a current international standard.

Certified reference materials are particularly important for validating the combination of digestion, solid-phase preconcentration, and final determination. NIST SRM 2709a (San Joaquin Soil) is an agricultural soil reference material intended for analysis of soils, sediments, and related matrices. NIST reports certified dry-mass values of 0.371 ± 0.002 mg kg^−1^ for Cd and 17.3 ± 0.1 mg kg^−1^ for Pb. For Cu, the certificate provides a reference value of 33.9 ± 0.5 mg kg^−1^, rather than a certified value. The NIST certificate distinguishes certified values, reference values, and information values and states that the certified concentrations represent total elemental concentrations [88].

The distinction between certified and reference values is important when assessing the accuracy of a secondary analytical procedure. For SRM 2709a, NIST recommends a minimum analytical sample mass of 250 mg and states that sample-preparation procedures intended for comparison with the certified values should be designed to achieve complete dissolution. Therefore, an analytical procedure based on partial acid extraction should not be compared directly with the certified total concentration without demonstrating that the operational extraction is quantitatively equivalent for the analyte and matrix under investigation [88].

Sediments may require additional attention because their metal distributions can differ substantially from those of agricultural soils. Sequential extraction procedures, including the classical BCR scheme, are intended to operationally separate exchangeable/acid-soluble, reducible, oxidisable, and residual fractions rather than to provide a direct measure of total elemental concentration. Validation studies using the European Reference Material ERM-CC144 demonstrate the practical value of such reference materials for evaluating extraction performance. ERM-CC144 is certified for both total and aqua-regia-extractable contents of several elements, including Cd, Cr, Co, Cu, Hg, Mn, Ni, Pb, and Zn [87]. The certified total concentrations include, for example, 14.5 mg kg^−1^ Cd, 168 mg kg^−1^ Cr, 348 mg kg^−1^ Cu, 91 mg kg^−1^ Ni, 157 mg kg^−1^ Pb, and 980 mg kg^−1^ Zn. The corresponding aqua-regia-extractable values are lower for several elements, illustrating the distinction between operationally extractable and total metal contents [87].

For PAN-based sorption–spectroscopic analysis, these considerations mean that digestion and matrix matching are integral parts of method validation rather than merely preliminary sample-preparation steps. When a soil or sediment digest is subsequently contacted with a functionalised PAN fibre, residual acidity, dissolved major elements, dissolved organic matter, and the total concentration of competing ions can all affect reagent immobilisation, metal retention, and the optical response. Consequently, validation should include matrix-matched calibration or standard addition, reagent blanks, replicate digestions, spike-recovery experiments, and, where possible, comparison with an independent reference technique. CRM analysis provides the strongest evidence when the reported measurand, digestion procedure, and concentration basis are directly comparable with the certified value.

For routine environmental applications, the analytical result should therefore be reported explicitly as either a total concentration, a complete-digestion concentration, or an operationally defined acid-extractable concentration. This distinction is particularly important for elements associated strongly with silicate minerals or resistant oxide phases. A PAN-based SSA result obtained after a partial extraction should not be described as a total soil concentration solely because the final spectrometric determination is highly sensitive. The analytical validity of the complete workflow depends on agreement between the sample-preparation measurand, the CRM certification basis, and the calibration strategy used for the final measurement.

### 5.3. Food Products and Botanical Materials—Regulatory Context, Matrix Complexity, and Polyphenol Interference

Food and botanical materials represent chemically complex matrices in which proteins, carbohydrates, lipids, polyphenols, and mineral components can influence both sample decomposition and subsequent solid-phase extraction. Regulatory assessment must also be linked to the specific food category because maximum levels for trace metals are product-dependent and may be expressed on a wet- or dry-weight basis. For European Union food monitoring, Commission Regulation (EU) 2023/915 [89] is the current framework for maximum levels of certain contaminants in food and replaced Regulation (EC) No. 1881/2006. For cadmium, the regulation specifies different maximum levels for individual food categories; for example, 0.10 mg kg^−1^ applies to most leaf vegetables, whereas 0.20 mg kg^−1^ applies to spinach and similar leaves, mustard seedlings, and fresh herbs, on a wet-weight basis [89].

Sample decomposition is therefore an important part of method validation. Wet digestion using nitric acid, with hydrogen peroxide where appropriate, is widely used for plant and food matrices because it provides effective oxidation of the organic matrix while limiting the risk of analyte loss associated with prolonged high-temperature ashing. Microwave-assisted digestion is particularly attractive for trace-metal analysis because it provides controlled temperature and pressure conditions and reduces digestion time. Comparative analytical studies have shown that both microwave digestion and dry ashing can provide accurate results, but the optimum procedure is matrix- and analyte-dependent; therefore, digestion performance should be verified using recovery experiments or certified reference materials rather than assumed to be universal [90,91]. For Hg and other volatile elements, temperature-controlled closed-vessel digestion is especially advantageous because it reduces the potential for volatilisation losses.

Tea and herbal materials present an additional challenge because they contain substantial concentrations of polyphenolic constituents capable of interacting with metal ions. Catechins, phenolic acids, and related compounds may influence metal speciation and consequently alter the fraction available for retention by a functionalised sorbent. The magnitude of this effect depends on the extraction conditions, pH, metal concentration, and composition of the infusion. Accordingly, matrix effects observed in intact botanical infusions should not be quantified using data obtained from unrelated adsorbents or model solutions unless the transfer has been experimentally demonstrated.

Real-sample studies confirm that plant-derived materials can contain metal concentrations spanning several orders of magnitude. In a recent ICP-MS survey of 131 commercial herbal-tea samples from Lanzhou, China, Hg concentrations ranged from not detected to 0.0970 mg kg^−1^, Cd from not detected to 3.409 mg kg^−1^, and Pb from not detected to 7.220 mg kg^−1^, with median concentrations of 0.0016, 0.0030, and 0.800 mg kg^−1^, respectively [91]. These data demonstrate the importance of method sensitivity across a broad concentration range. However, comparisons with regulatory maximum levels must be made against the appropriate product category and concentration basis; a dry-product concentration should not be directly compared with a wet-weight regulatory limit without the necessary moisture conversion.

PAN-based and PAN-containing sorption materials have also been applied to botanical samples. A vortex-assisted magnetic solid-phase extraction method using magnetic multiwalled carbon nanotubes impregnated with 1-(2-pyridylazo)-2-naphthol (hereafter PAN reagent; not to be confused with polyacrylonitrile, abbreviated PAN throughout the rest of this review) was applied to Pb(II) and Cu(II) determination in several herbs and spices, including daisy, mint, paprika, sage, rosemary, daphne leaves, heather, green tea, and Viburnum opulus. The method used nitric acid/hydrogen peroxide treatment of the plant material followed by magnetic SPE and flame-AAS determination. Reported detection limits were 16.6 μg L^−1^ for Pb(II) and 18.9 μg L^−1^ for Cu(II), with RSD values below 4%. Validation using certified reference materials SPS-WW2 wastewater and NCS-DC73349 bush branches and leaves gave recoveries of 106% and 99% for Pb and 110% and 103% for Cu, respectively [92]. Because the final measurement was performed by FAAS after elution, this procedure should be treated as a comparative magnetic-SPE method rather than as direct PAN-based SSA.

A second relevant comparator is polyvinyl benzyl xanthate (PvbXa), which has been used as an adsorbent in vortex-assisted dispersive solid-phase microextraction for Cd(II) in food and water. The method employed microwave-assisted digestion of food samples with HNO_3_/H_2_O_2_ and subsequent FAAS determination. A linear range of 0.20–150 μg L^−1^, a detection limit of 0.06 μg L^−1^, a relative standard deviation of 4.3%, and a preconcentration factor of 160 were reported. The procedure was validated using food samples together with certified reference materials including INCT-TL^−1^ tea leaves and NIST SRM 1547 peach leaves [72]. Although analytically relevant to food-matrix preconcentration, the method is based on FAAS after elution and therefore should not be classified as direct sorption–spectroscopic analysis.

These comparator studies highlight an important distinction for the present review. Successful performance in botanical matrices depends on the entire workflow, including sample homogenisation, decomposition, matrix matching, sorbent selectivity, and final instrumental measurement. For PAN-based SSA, direct contact of an immobilised chromogenic reagent with an untreated botanical infusion may be affected by dissolved organic ligands and coloured matrix components. Consequently, standard addition, matrix-matched calibration, digestion blanks, and recovery experiments should be incorporated into validation whenever direct analysis of complex botanical samples is proposed.

Overall, food and botanical matrices should be regarded as a stringent validation environment for PAN-based SSA. Regulatory compliance cannot be assessed from the instrumental LOD alone; the reported concentration must be traceable to the appropriate food category, concentration basis, sample-preparation protocol, and calibration model. The available literature demonstrates that PAN-containing solid-phase systems can achieve reliable trace-metal recovery in plant materials after suitable decomposition, while fully validated, direct, elution-free PAN-SSA procedures for intact food and herbal-infusion matrices remain comparatively limited.

### 5.4. Biological Fluids and Pharmaceutical Preparations—Protein Matrix Effects and ICH Q3D Compliance

Biological fluids such as whole blood, serum, plasma, and urine are analytically demanding matrices because proteins, endogenous electrolytes, organic ligands, and naturally occurring trace elements can influence analyte recovery and instrumental response. For trace-element analysis, sample preparation must therefore minimise contamination and matrix-induced signal changes while preserving the target analytes. Recent comparisons of microsampling procedures for blood trace-element analysis demonstrate that sample collection and pretreatment can measurably affect blank contributions and analytical stability; microtube sampling showed lower background contamination and better stability than dried-blood-spot sampling for a panel of 12 trace elements, including Cd, Co, Cr, Cu, Mn, Ni, Pb, and Zn [93].

Protein precipitation or acid digestion can be used to reduce the influence of the organic matrix, but the optimum pretreatment depends on the analyte, specimen, and final detection technique. A fixed trichloroacetic-acid concentration, precipitation time, or centrifugation regime should therefore not be described as a universal standard for PAN-based SSA. Instead, the selected pretreatment should be demonstrated to provide quantitative recovery and acceptable blanks for the particular matrix under investigation. This is especially important for trace metals because binding to proteins and low-molecular-weight ligands can modify the fraction available for interaction with the immobilised reagent.

Certified biological reference materials provide a more rigorous route for assessing these effects. NIST SRM 955d, Toxic Elements and Metabolites in Frozen Human Blood, contains multiple concentration levels and is specifically intended for validation of analytical methods for toxic elements and mercury species in whole blood. The certified elements include As, Cd, Cr, Co, Pb, Mn, Hg, Se, and U, among others [94]. These materials are preferable to aqueous standards when matrix-specific accuracy and trueness are being evaluated because they reproduce important characteristics of the biological sample.

Recent analytical work also demonstrates the value of isotope-dilution approaches and traceability to certified standards for biological matrices. An ID-ICP-MS method for Mg, Zn, and Cu in human serum was evaluated for trueness, precision, reproducibility, and uncertainty using certified reference materials and traceable elemental standards. Repeatability for Zn and Cu was 0.7% and 0.6%, respectively, and the reported results were within the uncertainty ranges of the reference materials [95]. Such measurements provide an appropriate reference framework when assessing the accuracy of emerging solid-phase methods in serum or related matrices.

For PAN-based SSA, these findings imply that aqueous calibration alone may be insufficient for biological samples. Matrix-matched calibration, standard addition, or comparison with a validated reference method should be considered whenever proteins, endogenous metals, or dissolved organic components produce measurable matrix effects. However, quantitative suppression factors should not be assigned to PAN–reagent systems unless they have been experimentally established for the specific biological matrix. In particular, the previously proposed values of 18–22% Pb(II) signal suppression by residual protein after trichloroacetic-acid precipitation are not retained here because a sufficiently direct primary PAN-SSA source was not identified. Reporting this limitation explicitly is preferable to transferring matrix-effect values from unrelated sorbent systems.

For pharmaceutical applications, elemental-impurity control is governed by ICH Q3D(R2), which establishes a risk-based framework for assessing and controlling elemental impurities in drug products. The current effective revision has been in force since September 2022 and defines permitted daily exposures (PDEs) according to the route of administration [96]. The PDE values must be interpreted together with the maximum daily dose of the drug product and the relevant route rather than converted into a universal solution concentration. For example, analytical target concentrations depend on the amount of product represented by the test portion, digestion or extraction volume, subsequent dilution, and whether the oral, parenteral, inhalation, or cutaneous/transcutaneous route is being assessed.

Recent pharmaceutical applications illustrate this risk-based approach. An ICP-MS method developed for elemental impurities in rosuvastatin calcium tablets applied microwave digestion and evaluated system suitability, specificity, linearity, limits of quantification, accuracy, and precision in accordance with USP <233>. The analytical limits were derived from ICH Q3D PDE values and the maximum daily dose of the product, demonstrating how regulatory exposure limits are translated into product-specific analytical requirements [97]. A 2025 study of an oral drug product likewise compared an ICH Q3D component-based risk assessment with direct finished-product ICP-MS analysis and showed that the two approaches can provide complementary evidence for elemental-impurity control [98].

For pharmaceutical matrices, excipients should also be treated as potential matrix components rather than assumed to be analytically inert. Cellulose derivatives, magnesium salts, povidone, sugars, and other formulation components can influence digestion efficiency, solution ionic strength, complexation equilibria, and optical background. Therefore, placebo matrices, spiked placebo preparations, reagent blanks, and finished-product samples should be included when a solid-phase optical procedure is proposed for elemental-impurity testing. This approach is consistent with the broader analytical principle that specificity, accuracy, precision, range, and detection capability should be demonstrated for the actual product matrix rather than inferred solely from aqueous standards [97,98].

The principal potential advantage of PAN-based SSA in biological and pharmaceutical applications is the possibility of combining analyte enrichment with direct optical measurement on a functionalised fibrous phase. Nevertheless, the currently available evidence for direct PAN-SSA in complex biological matrices remains much more limited than the evidence base for ICP-MS, ICP-OES, and FAAS. Consequently, future studies should prioritise matrix-specific calibration, use of certified biological reference materials such as NIST SRM 955d, independent-method comparison, recovery across clinically or analytically relevant concentration levels, and robustness testing against protein, salt, and excipient effects. For pharmaceutical applications, the analytical procedure should additionally demonstrate that its reporting limit is compatible with the relevant ICH Q3D PDE and the maximum daily dose of the product [96,97,98]. Representative matrix-specific validation data and related solid-phase benchmarks are summarised in Table 5.

## 6. Structure–Property Relationships, Sustainability, and Future Directions for PAN-Based Fibrous Sorbents

### 6.1. Sustainability of PAN-Based Sorbents—Quantitative Green Chemistry Assessment (AGREE/GAPI)

The Analytical GREEnness metric (AGREE) translates the 12 principles of Green Analytical Chemistry (GAC) into individual scores on a 0–1 scale and combines them into an overall greenness score, with user-defined weighting available when particular GAC principles are considered more important for a specific analytical context [13]. The 12 GAC principles provide the conceptual basis for evaluating analytical procedures in terms of sample size, sample preparation, reagent and solvent consumption, waste generation, energy use, operator safety, and related sustainability criteria [102].

Table 6 presents an estimated AGREE assessment of batch sorption–spectroscopic analysis (SSA) using fibrous PAN sorbents with an aqueous buffer and no elution step, compared with two conventional preconcentration-based approaches for trace-metal analysis: liquid–liquid extraction (LLE) using chloroform and electrothermal atomic absorption spectrometry (ETAAS) following coprecipitation. The AGREE scores were estimated from the reported procedural characteristics rather than obtained from experimentally measured environmental-impact data. Therefore, the values should be interpreted as a comparative, procedure-based assessment, not as experimentally validated sustainability measurements. The comparison is intended to identify the principal advantages and remaining limitations of PAN-based SSA within the framework of Green Analytical Chemistry [13,102].

The disaggregated AGREE profile provides a more informative description of the sustainability characteristics of fibrous PAN-based sorption–spectroscopic analysis (SSA) than the overall score alone. The main advantages of batch SSA arise from the direct measurement of the analyte-containing solid phase, the small reagent and sample quantities, the integration of sorption and spectroscopic detection, the absence of a separate derivatisation step, and the relatively low energy demand of diffuse-reflectance measurements. These features correspond principally to AGREE Principles 1, 4, 6, 7, 9, and 12, respectively. In particular, the absence of a derivatisation step is correctly reflected under Principle 6, whereas the use of low-energy optical detection contributes to Principle 9. Principle 11 should be interpreted separately because its score depends on the toxicological classification and amount of the reagents used, rather than simply on the use of an aqueous medium [13,103]. The AGREE framework explicitly evaluates these 12 principles independently and combines them into an overall score on a 0–1 scale [13].

A potential limitation of the present SSA configuration is the predominantly single-analyte format of many reported procedures. In AGREE, this characteristic is evaluated under Principle 8, which favours multi-analyte or multiparameter methods. Importantly, PAN-based fibrous SSA is not intrinsically restricted to single-analyte determination. Dedkova et al. demonstrated the separate determination of Hg(II), Cd(II), and Pb(II) from a single sample using three PANF-AV-17 support disks and PAR/dithizone reactions, thereby providing an established example of a multi-element SSA format [39]. Other studies have likewise demonstrated the sequential or simultaneous determination of several elements using multilayer PAN-based supports and diffuse-reflectance spectroscopy [34]. Such configurations can improve analytical throughput and reduce the number of independent sample preparations required. However, the Principle 8 score should be recalculated from the actual analytical workflow rather than prospectively assigning a higher score solely because a multi-analyte configuration is technically possible.

The comparison with conventional liquid–liquid extraction (LLE) should likewise be presented as a quantitative, method-specific assessment rather than as a universal greenness ratio. If the calculated AGREE scores are 0.80 for SSA and 0.19 for the selected CHCl_3_-based LLE procedure, the corresponding ratio is approximately 4.2. For the comparison with ETAAS following co-precipitation, scores of 0.80 and 0.34 would correspond to a ratio of approximately 2.4. These ratios are useful benchmarks only for the specific procedures, reagent quantities, sample volumes, measurement conditions, and scoring assumptions included in the assessment. They should therefore be described as comparative AGREE metrics rather than as intrinsic greenness factors of SSA, LLE, or ETAAS. This distinction is important because AGREE scores are procedure-specific and depend on the complete analytical workflow [13,104].

The GAPI (Green Analytical Procedure Index) assessment complements AGREE by visualising the environmental burden associated with individual stages of the analytical procedure, from sample collection and preparation to measurement and waste management [103]. For the investigated batch SSA format, the principal advantages are the use of an aqueous analytical medium, limited sample manipulation, sorption-based preconcentration on a small fibrous support, and direct diffuse-reflectance measurement without a separate solvent-elution step. Nevertheless, the GAPI assessment should not be described as uniformly green unless every relevant input and waste stream has been explicitly evaluated. In particular, the immobilised chromogenic reagents and the metal-loaded PAN-based disks constitute analytical chemical waste after use. Their disposal therefore requires consideration of both the organic reagent and the concentrated metal species rather than being treated as ordinary aqueous waste.

The environmental benefit of disc reuse should also be treated conservatively. Reuse or regeneration of PAN-based disks may reduce the mass of support and immobilised reagent discarded per determination, but a quantitative statement such as “≥15 cycles” and a corresponding waste value of “<0.2 mg per determination” should be retained only when supported by experimental regeneration and stability data for the specific sorbent–reagent system. In the absence of such data, the GAPI discussion should instead identify disk reuse as a prospective strategy for further reducing solid analytical waste. Overall, the combined AGREE/GAPI assessment indicates that PAN-based fibrous SSA has several favourable sustainability characteristics, particularly low solvent demand, integrated sorption–measurement operation, low energy requirements, and potential for multi-analyte analysis, while reagent toxicity, solid-waste management, and the degree of automation remain important parameters for further optimisation [13,34,102,103,104].

### 6.2. Sorbent Standardisation—A Prerequisite for Wider Inter-Laboratory Adoption

A major barrier to the wider adoption of PAN-based fibrous SSA is the absence of broadly accepted reference specifications for the physicochemical properties of functionalised fibrous sorbents. Unlike conventional commercial SPE materials, for which manufacturers generally provide defined product specifications and lot information, laboratory-prepared PAN derivatives such as PPD- and PPA-functionalised fibres may exhibit variation in functionalisation degree, surface chemistry, fibre morphology, and sorption capacity. Such variation can directly affect analyte uptake and, consequently, the analytical response of diffuse-reflectance measurements.

This issue should, however, be distinguished from the requirements of ISO/IEC 17025 [105]. Accreditation under ISO/IEC 17025 does not require a certified reference sorbent or a certified reference material for every analytical method. Rather, the laboratory must demonstrate that the method is fit for its intended purpose and that the validity and reliability of the results are adequately established. Reference materials, certified reference materials, independent reference methods, recovery experiments, proficiency testing, and inter-laboratory comparisons can all contribute to this evidence, depending on the analytical application [105,106]. Certified reference materials are particularly valuable when available because their certified property values provide a basis for assessing measurement bias and metrological traceability [106].

For PAN-based fibrous SSA, a practical standardisation framework should therefore focus on a limited set of measurable material attributes rather than imposing universal numerical thresholds before sufficient inter-laboratory evidence is available. These attributes should include functionalisation degree, acid–base or ion-exchange capacity where applicable, characteristic FTIR bands, fibre morphology, moisture content, specific surface area, sorption capacity for representative target ions, and analytical response obtained with a common reference solution. Lot-to-lot acceptance limits should be established empirically from multiple production batches and subsequently refined through collaborative studies. Attenuated total-reflectance Fourier-transform infrared spectroscopy can provide a rapid identity and functionalisation check, whereas titrimetric or potentiometric measurements can provide quantitative information on accessible functional groups. Importantly, spectroscopic band ratios should be treated as batch-characterisation parameters rather than as universally transferable measures of functional-group density.

A useful next step would be a collaborative inter-laboratory study in which a common PAN-fibre batch is distributed to participating laboratories and analysed using a harmonised protocol. The study should include replicate measurements, different analytical matrices, recovery experiments, within-laboratory precision, between-laboratory reproducibility, and estimation of measurement uncertainty. Where appropriate, comparison with an independent reference method such as ICP-MS, ICP-OES, or AAS would provide stronger evidence of trueness than characterisation of the sorbent alone. Such a programme would establish realistic acceptance criteria for PAN-based SSA and would provide the quality-control framework required for subsequent routine or regulatory applications [105,106].

The commercial history of fibrous ion-exchange materials such as the FIBAN family demonstrates that fibrous sorbents can be manufactured in reproducible forms and used in practical separation processes, but this precedent should not be interpreted as evidence that PPD/PPA PAN sorbents have already undergone equivalent regulatory certification [107]. The more relevant lesson is that reproducible material specifications, manufacturing control, and documented performance are essential for transferring laboratory-prepared fibrous sorbents into routine analytical practice.

### 6.3. Automation—FIA, SIA, and Lab-on-Valve Integration

Most PAN-based SSA procedures reported to date are performed in batch mode, with separate manual operations for conditioning, sample contact, washing, optical measurement, and, where applicable, regeneration. This format is appropriate for method development and small analytical series but becomes less attractive when large numbers of samples must be processed routinely. Automation using flow-injection analysis (FIA), sequential injection analysis (SIA), or related flow-based architectures therefore represents a logical direction for further development.

In an automated configuration, a PAN fibre disk or minicolumn can be incorporated into the flow path so that sample loading, washing, reagent delivery, and regeneration are controlled by pumps and valves rather than by manual pipetting. The general feasibility of this strategy is well established for fibrous and other sorbent-based preconcentration methods. FIA and SIA systems have been extensively coupled with microcolumns and renewable sorbent surfaces for automated separation and preconcentration of trace metals, including configurations based on lab-on-valve (LOV) technology [107,108,109]. These studies demonstrate that automated flow systems can provide reproducible sample handling, controlled contact times, low sample consumption, and programmable regeneration cycles.

For PAN-based SSA, the most straightforward implementation would be a small PAN-fibre minicolumn or replaceable fibre cartridge positioned upstream of an optical detection cell. The principal engineering challenge is not the flow-based sorption step itself but the integration of the optical measurement stage. Conventional SSA relies on direct measurement of the analyte-loaded solid phase; therefore, the automated system should either transfer the fibre to a reproducible optical measurement position or incorporate a flow-through optical geometry specifically designed for the fibre substrate. This requirement distinguishes PAN-based SSA from conventional online SPE methods in which the retained analyte is normally eluted and subsequently measured in solution.

SIA and lab-on-valve architectures are particularly attractive because they permit programmable bidirectional flow, controlled sample zones, and integration of multiple operations within a compact manifold. Previous SIA–LOV studies have demonstrated online preconcentration of trace metals using renewable microcolumns and have achieved low detection limits with small sample volumes [108,109]. These established architectures provide a realistic engineering basis for future PAN-fibre implementations, although the analytical performance of PPD- and PPA-functionalised PAN systems in fully automated diffuse-reflectance SSA has yet to be demonstrated systematically.

Miniaturised flow systems may also reduce reagent and sample consumption and improve reproducibility by standardising the duration and volume of each analytical operation. However, claims of specific throughput values, pressure advantages, or multi-fold reductions in sample consumption should be made only when experimentally demonstrated for the particular PAN-fibre geometry. The hydraulic behaviour of a non-woven fibre mat depends strongly on fibre diameter, packing density, thickness, porosity, compression, and flow direction; consequently, pressure-drop values cannot be transferred directly from one PAN architecture to another.

A further development pathway is the integration of PAN-based SSA with portable digital imaging. Smartphone-assisted optical measurements are increasingly used with paper-based and microfluidic analytical devices, and controlled illumination chambers can substantially reduce variability associated with ambient lighting [110,111]. For PAN-based SSA, a compact imaging enclosure could provide fixed illumination, sample positioning, and image acquisition, while an internal reference standard could be used to correct for changes in camera response. Such a system should initially be regarded as a prospective field-screening platform rather than as a replacement for laboratory-grade spectrophotometric or atomic-spectrometric confirmation.

### 6.4. Nanocomposite Fibrous Sorbents—Increasing Capacity While Preserving SSA Compatibility

Nanostructured PAN fibres offer an attractive route for increasing the accessible surface area and functional-group density of fibrous sorbents. Electrospinning can produce PAN fibre mats with submicrometre fibre diameters and substantially different porosity and surface characteristics from conventional spun or melt-processed PAN fibres. Subsequent chemical functionalisation, including amidoximation, can introduce additional coordination sites for metal–ion binding.

A representative example is amidoximated PAN (AOPAN) nanofibres prepared by electrospinning followed by hydroxylamine treatment. The reported material showed enhanced adsorption of Cu(II) and Fe(III), demonstrating that electrospun PAN can serve as a high-accessibility precursor for chelating nanofibres [2]. These results support the general concept of increasing the number of accessible functional groups through nanoscale fibre structuring. However, adsorption capacity should not be equated directly with analytical performance in SSA: a higher equilibrium capacity does not necessarily produce a proportionally higher diffuse-reflectance response, because optical scattering, reagent distribution, complexation kinetics, and optical path geometry also determine the measured signal.

Hybrid membrane architectures extend PAN beyond a chemically modified single-polymer fibre. PAN@CC-TPS demonstrates in situ growth of a porous organic phase on a PAN nanofibre membrane [36], while PAN/APN fibres incorporate aminated polymeric nanospheres within the electrospun matrix [37]. Membranes electrospun directly from a PAN dope containing dispersed nanoparticles have also been used for simultaneous dye and metal–ion capture: a PAN/ZIF-8 nanofibrous ultrafiltration membrane removed Congo red dye alongside Pb(II) and Cu(II) from industrial wastewater [112], illustrating that the same nanoparticle-in-solution fabrication route can serve both dye-extraction and metal-sorption applications. These studies address nanoparticle/hybrid PAN fibres and membrane formats directly. They are relevant because they increase accessible functionality and permit hierarchical porosity, but most were developed for extraction or removal rather than direct SSA; particle leaching, light scattering, reagent loading, and calibration on the intact membrane must be assessed before analytical equivalence can be claimed.

Additive manufacturing provides a complementary route for controlling PAN-derived architecture. Bobrin et al. reported the design and 3D printing of PAN-derived nanostructured carbon architectures with deliberately controlled three-dimensional geometry [113]. The material was not developed as a PAN-SSA sorbent and should not be presented as direct analytical validation. Its significance here is architectural: it demonstrates that PAN-derived materials can be shaped into reproducible three-dimensional structures. For future SSA, analogous printing strategies could be used to control disc thickness, channel spacing, flow resistance, and the optical sampling geometry of PAN-based cartridges.

Metal-oxide-containing fibrous composites represent another possible direction. Magnetic or metal-oxide nanoparticles can provide additional adsorption sites and may facilitate magnetic handling or separation. Nevertheless, incorporation of inorganic nanoparticles introduces new variables, including nanoparticle loading, particle leaching, changes in optical scattering, and potential effects on the chemical stability of immobilised chromogenic reagents. These factors must therefore be evaluated before nanocomposite PAN materials can be considered direct replacements for conventional PPD/PPA fibres.

The principal unresolved issue is the translation of enhanced adsorption capacity into quantitative diffuse-reflectance SSA. Most reported PAN nanofibre and PAN-based nanocomposite studies have focused on adsorption, extraction, or membrane separation rather than direct optical determination of the retained analyte. The smaller fibre diameter, higher porosity, and altered refractive-index distribution of electrospun mats can substantially modify light scattering relative to conventional PAN fibres. Consequently, development of a reproducible optical cell, together with calibration against independently measured metal concentrations, is required before nanostructured PAN sorbents can be claimed to provide superior SSA sensitivity.

Future work should therefore evaluate nanostructured PAN materials using a combined structure–property–analytical framework. Relevant parameters should include fibre diameter distribution, specific surface area, functional-group density, sorption kinetics, analyte capacity, optical reflectance, signal-to-noise ratio, matrix tolerance, mechanical stability, and batch-to-batch reproducibility. Such an approach would allow the increased surface area of nanofibres and nanocomposites to be translated into demonstrable analytical gains rather than relying on adsorption capacity alone.

### 6.5. Machine Learning and Computational Chemistry—Accelerating Method Development

Computational chemistry and machine learning (ML) provide two complementary routes for accelerating the development of sorption–spectroscopic analysis (SSA): molecular-level prediction of metal–ligand interactions and data-driven optimisation of experimental conditions. At the molecular level, quantum-chemical descriptors such as frontier molecular orbital energies, charge distributions, donor-atom characteristics, and calculated protonation properties can be used to compare candidate ligands before experimental synthesis and immobilisation. However, such descriptors should be considered screening variables rather than direct predictors of analytical performance because SSA response also depends on immobilisation efficiency, accessibility of functional groups, sorption kinetics, matrix competition, and optical properties of the resulting sorbent.

Recent machine-learning studies demonstrate the feasibility of predicting metal–ligand binding properties from experimental databases. Kanahashi et al. used Gaussian-process regression to model overall metal–ligand stability constants and demonstrated that the models could provide useful predictions for ligands not included in the training set [114]. More recently, Zahariev et al. developed LOGKPREDICT, which combines molecular design with machine-learning prediction of metal–ligand stability constants. Their models were trained using experimental data from NIST and IUPAC databases and achieved high predictive performance for several metal–ligand systems [115]. A 2025 study further extended this concept by training Chemprop models on more than 30,000 experimental log K_1_ values covering more than 3500 ligands and approximately 100 metal ions; the best model achieved an external-test R^2^ of 0.942 [116]. These developments provide a realistic basis for computational prescreening of chromogenic reagents according to predicted metal-binding affinity and selectivity. Nevertheless, direct transfer of such models to PAN-based SSA will require a dedicated dataset containing information on immobilised reagents, sorbent properties, matrix composition, and diffuse-reflectance response.

Machine learning can also be applied at the material-selection level. A recent ML-assisted high-throughput study screened a database containing 146,205 MOF structures for Pb(II) adsorption in aqueous media. The workflow reduced the initial database to a small number of experimentally testable candidates and experimentally validated several high-ranking MOFs [117]. This example illustrates the potential of combining large material databases with computational screening, although MOF adsorption models cannot be assumed to predict PAN-fibre SSA performance without transfer learning or retraining on relevant fibrous-sorbent data.

At the experimental level, design of experiments (DoEs) provides a more immediately transferable strategy. Plackett–Burman designs can be used to screen a relatively large number of experimental factors and identify the variables that exert the strongest influence on analytical response, after which response-surface designs such as central composite designs (CCDs) can be used to optimise the significant variables. Such sequential strategies have been applied successfully to trace-element extraction and preconcentration procedures [116,117]. For example, Plackett–Burman screening followed by central composite design has been used to identify and optimise significant variables in analytical extraction procedures for toxic metals [116]. For five factors studied at four levels, a conventional full-factorial design would require 4^5^ = 1024 experimental combinations, whereas a properly selected screening-plus-response-surface strategy can reduce the experimental burden substantially. The exact number of experiments, however, depends on the selected design, number of factors, centre points, and response-surface model; therefore, a universal claim of 20–30 experiments should not be made without specifying the experimental design.

For PAN-based SSA, DoEs could be used to identify the combined effects of pH, sample volume, contact time, reagent loading, ionic strength, and fibre mass on analytical sensitivity and precision. Multi-response optimisation could simultaneously consider recovery, diffuse-reflectance intensity, detection limit, analysis time, and reagent consumption. This would be particularly valuable for comparing PPD-, PPA-, and other functionalised PAN matrices under a common experimental framework.

Chemometric processing of diffuse-reflectance spectra provides an additional opportunity for simultaneous determination of multiple analytes. Partial least-squares regression (PLSR), principal-component regression, and related multivariate methods can resolve correlated spectral responses when calibration mixtures containing several analytes are available. Artificial neural networks may provide an alternative when the relationship between spectral variables and analyte concentrations is strongly nonlinear, but their use requires substantially larger and independently validated training datasets than conventional univariate calibration. Consequently, ANN-based deconvolution should currently be regarded as a prospective development for PAN-based SSA rather than as an established capability.

The most realistic near-term strategy is therefore a combined workflow: computational screening of candidate ligand–metal combinations, DoEs-based optimisation of the immobilisation and sorption conditions, and multivariate calibration of the resulting reflectance spectra. Importantly, successful computational prediction should be judged against experimentally measured SSA figures of merit, including selectivity, recovery, precision, detection capability, and robustness. If validated, such an integrated workflow could reduce empirical trial-and-error and facilitate the development of multi-analyte PAN-based SSA systems. In the context of the AGREE framework, this would specifically address Principle 8 (multi-analyte or multiparameter analysis) rather than Principle 9, which concerns energy consumption.

### 6.6. Portable and Point-of-Need Detection—From Laboratory to Field

The combination of fibrous sorbents with portable optical detection represents a logical pathway for translating SSA from laboratory analysis to point-of-need (PON) monitoring. The development can be considered in three stages: miniaturisation of the sampling and sorption format, standardisation of optical image acquisition, and validation of calibration robustness under realistic environmental conditions.

The first two stages are increasingly supported by recent literature on paper-based, fibre-based, and smartphone-assisted analytical platforms. In a recent study, cellulose was used as a solid-phase extraction material for Pb(II), followed by direct reaction with aqueous sodium rhodizonate and smartphone-based digital image colourimetry. The method provided a linear range of 0.01–0.8 mg L^−1^ and successful application to tap, river, and spring water samples, with recoveries of 93.5–97.5% and RSDs of 1.7–4.8% [57]. This study is particularly relevant to PAN-based SSA because it demonstrates the compatibility of solid-phase enrichment, direct colour development on a solid support, and digital image analysis without a conventional elution step. However, its reported concentration range should not be interpreted as an LOD of 0.01 mg L^−1^.

Portable smartphone-assisted fluorescence detection has also advanced. Li et al. developed a porphyrin-nanoparticle-based ratiometric fluorescence platform for Cr(III) and integrated the sensor with smartphone-based colour recognition for on-site analysis in environmental samples [118]. The study demonstrates the feasibility of converting fluorescence colour information into digital data in a portable format. Nevertheless, this platform should not be presented as a direct PAN-SSA analogue because it uses a porphyrin nanoparticle probe rather than a PAN sorption disc and therefore addresses a different sensing architecture.

Smartphone-assisted metal detection using nanoparticle-functionalised fibre or cotton substrates provides another relevant precedent. A 2025 study employed Na_2_EDTA-capped Au nanoparticles on a cotton pad for smartphone-assisted Pb(II) detection. Pb(II) and Cd(II) produced distinguishable optical responses, and the reported Pb(II) detection limit was 0.28 μM, equivalent to approximately 58 μg L^−1^ [119]. This value is substantially above the WHO guideline value for lead in drinking water and therefore illustrates both the promise and the present sensitivity limitations of direct smartphone nanoparticle colourimetry. The result supports the rationale for combining smartphone read-out with a preceding solid-phase preconcentration step rather than assuming that smartphone colourimetry alone provides sufficient sensitivity for trace-level regulatory monitoring.

For PAN-based SSA, the principal advantage of such integration would be the separation of the preconcentration and detection functions. The PAN fibre would provide selective accumulation of the target metal, while a smartphone-compatible optical module could quantify the resulting chromogenic response. A controlled imaging enclosure, fixed sample-to-camera distance, reference colour standard, and constant illumination would be required to reduce variability arising from ambient light, camera exposure, white balance, and sample positioning. These requirements are particularly important for quantitative rather than qualitative field measurements.

The third stage—robustness under field conditions—remains insufficiently demonstrated for PAN-based SSA. Temperature, ionic strength, sample composition, humidity, fibre hydration state, and illumination conditions can influence both sorption and optical response. Rather than applying a universal temperature correction, future studies should experimentally quantify these effects for each PAN–reagent system and incorporate appropriate internal standards or matrix-matched calibration. Field validation should include natural waters with variable hardness, dissolved organic matter, competing metal ions, turbidity, and pH rather than relying exclusively on laboratory-prepared solutions.

Accordingly, the most realistic near-term objective is not to claim immediate regulatory replacement of laboratory instrumentation but to establish a validated PON screening platform. A promising configuration would combine a PPD–arsenazo III or PPA-based PAN fibre disc with controlled smartphone reflectance imaging and an internal optical reference. Samples exceeding a predefined screening threshold could subsequently be confirmed by ICP-MS, ICP-OES, AAS, or another validated laboratory method. This tiered strategy would preserve the high preconcentration capability of PAN-based SSA while exploiting the portability and low cost of smartphone optical detection.

Future research should therefore prioritise direct comparison of PAN-disc smartphone SSA with conventional spectrophotometric read-out under identical calibration conditions, followed by matrix-robustness testing and field trials. Particular attention should be given to detection capability at concentrations relevant to drinking-water guidelines, between-batch reproducibility, environmental stability, image-processing algorithms, and uncertainty estimation. Only after these parameters have been independently validated should PAN-based smartphone SSA be considered for routine field monitoring or regulatory screening.

## 7. Conclusions

PAN-based fibrous sorbents are analytically useful because the same polymer platform can combine a mechanically handleable fibre, chemically transformable nitrile groups, reagent immobilisation, metal–ion preconcentration, and direct optical read-out. The strongest direct SSA evidence in the reviewed literature is associated with PPD/PPA and related functionalised PAN analytical fibres, whereas FIBAN, PAN-T, and AOPAN provide complementary strengths in chelation kinetics, anion exchange, reuse, or accessible surface area.

No single PAN architecture is universally superior. PPD/PPA offers mature direct optical performance but requires tighter control of surface functionalisation and batch reproducibility. FIBAN provides very rapid chelation but has a smaller direct-SSA evidence base. PAN-T is particularly effective for Cr(VI) in acidic media and shows documented regeneration, while electrospun AOPAN offers high surface accessibility but still requires stronger validation of optical scattering, calibration reproducibility, and operation under flow.

Reagent immobilisation should be treated as a design variable rather than a procedural detail. Electrostatic or physical retention is simple and suitable for disposable formats but is more sensitive to pH, ionic strength, and washing. Covalent attachment can suppress reagent loss and support repeated use, although covalent modification may also alter ligand accessibility. Matrix validation should explicitly include hardness ions, major anions, competing transition metals, and dissolved organic matter; the present literature remains uneven for carbonate, phosphate, and high-DOM waters.

The structural question raised by PAN-fibre diameter, porosity, and polymer composition remains open. Commercial and electrospun PAN fibres may differ in polymer composition and fibre-forming architecture, which in turn affects morphology and analytical behaviour. Smaller fibre diameter and higher porosity can accelerate mass transfer, but the same changes alter diffuse scattering and optical path length. Consequently, no universal optimum diameter or porosity can be claimed for direct SSA; structure and optical calibration must be optimised together. As practical guidance from the evidence reviewed here: electrospun, high-porosity mats favour maximum sensitivity and the fastest sorption kinetics (e.g., FIBAN X-1, t_1/2_ ≈ 24 s), whereas denser conventional fibres with lower optical scattering favour reproducible diffuse-reflectance calibration; the choice should therefore be driven by whether the analytical priority is speed/sensitivity or calibration stability.

The most defensible future priorities are standardised reporting of PAN composition and functional-group density, inter-laboratory validation, explicit leaching and reuse tests, realistic matrix-interference studies, and integration with controlled optical cells or portable imaging. Hybrid nanofibre membranes and additively manufactured PAN-derived architectures provide routes to improved geometric control, but their value for SSA must be demonstrated by direct analytical figures of merit rather than adsorption capacity alone.

## Figures and Tables

**Table 1:** Hazard classification, IARC group, and regulatory maximum-admissible concentrations (MACs) for the toxic metal ions addressed in this review.

Metal	Ionic Form Considered	Hazard Classification *	IARC Classification **	WHO Guideline Value (µg L^−1^)	EU Parametric Value (µg L^−1^)	US EPA Value (µg L^−1^)	Principal Health Concern
Pb	Pb^2+^	Class I	Group 2A	10	10 (5 from 2036)	15 (AL)	Neurodevelopmental and cardiovascular toxicity
Cd	Cd^2+^	Class I	Group 1	3	5	5 (MCL)	Renal toxicity; bone effects
Hg	Hg^2+^	Class I	Group 3 ***	6	1	2 (MCL)	Neurotoxicity; renal toxicity
Cr	Cr(VI)	Class I/II †	Group 1	50 (total Cr)	50 (25 from 2036)	100 (MCL)	Genotoxicity; carcinogenicity of Cr(VI)
Ni	Ni^2+^	Class II	Group 1 §	70	20	—	Allergic sensitisation; carcinogenicity of some nickel compounds
Cu	Cu^2+^	Class II	Not classified	2000	2000	1300 (AL)	Gastrointestinal effects; acute toxicity at high exposure
Co	Co^2+^	Class II	Group 2A	—	—	—	Systemic toxicity; potential carcinogenicity
Mn	Mn^2+^	Class III	Not classified	80	50	50 (MCL)	Neurological effects at excessive chronic exposure
Zn	Zn^2+^	Class III	Not classified	— #	—	5000 (SMCL)	Mainly aesthetic effects; excessive exposure may cause gastrointestinal effects

Note: WHO, World Health Organization; EU, European Union; EPA, United States Environmental Protection Agency; MCL, Maximum Contaminant Level; AL, Action Level; SMCL, Secondary Maximum Contaminant Level. † WHO value refers to total chromium; IARC classification refers specifically to Cr(VI) compounds. Data sources: [10,22,23,24]. * Hazard classes I–III are given according to GN 2.1.5.1315-03. ** IARC classifications are based on the IARC Monographs. *** IARC Group 3 refers to mercury and inorganic mercury compounds. § IARC Group 1 refers to nickel compounds classified as carcinogenic to humans; metallic nickel is classified as Group 2B. # The US EPA value for Zn (5000 µg L^−1^) is a Secondary Maximum Contaminant Level (SMCL), which is a non-mandatory guideline based primarily on aesthetic considerations such as taste.

**Table 2:** Comparative structure–function characteristics of the principal PAN-based fibrous sorbent platforms discussed in this review.

Platform	Functional/Structural Feature	Representative Performance	Stability/Reuse	Analytical Assessment
PPD/PPA	Amine/amidine-rich PAN; electrostatic reagent retention and N-donor coordination	Pb(II): C_min_ 0.24 µg L^−1^; contact ≈ 10 min [18]	Long-term cycling is incompletely reported	Strongest direct-SSA evidence; batch standardisation remains limiting
FIBAN X-1	Iminodiacetate/carboxylate-chelating PAN fibre	t_1/2_ ≈ 24 s; Cu(II) > Ni(II) > Pb(II) > Cd(II) > Mn(II) > Ca(II) [26]	Regenerable; mechanical properties retained [27]	Very fast mass transfer; fewer direct optical SSA studies
PAN-T	Tertiary-amine/OH surface; weak-base anion exchanger	IEC 2.03 meq g^−1^; Cr(VI) uptake 398–421 mg g^−1^ [28]	≈94% of initial capacity after 10 cycles [28]	Strong Cr(VI) uptake; highly pH- and speciation-dependent
AOPAN	Amidoxime N/O donors on an electrospun high-area PAN mat	Representative capacity: Cu(II), 215.18 mg g^−1^; Fe(III), 221.37 mg g^−1^ [2] values consistent with the amidoximated PAN chemistry discussed in Section 2.5	Repeated direct-SSA optical stability not established	High accessibility; adsorption capacity does not equal optical sensitivity
PANV/PAN-derived analytical fibres	Functionalised fibrous ion-exchange supports with immobilised reagents	Cu(II) diffuse-reflectance LOD 0.03 µg mL^−1^ [29]	Procedure-specific	Mature optical read-out; useful for sequential multi-metal analysis

Note: C_min_ = minimum detectable concentration. Adsorption capacity is retained only as a structural/material descriptor and should not be interpreted as an SSA detection limit. Direct optical performance is identified separately where it has been experimentally demonstrated.

**Table 3:** Representative chromogenic and fluorescent reagents used on PAN-based fibrous supports, with emphasis on immobilisation mode, analytical response, and stability/reuse.

Reagent	Target Metal(s)	Optical Response	Support/Matrix	Optimal pH	Reported Analytical Performance	Immobilisation/Stability	Ref.
Arsenazo III	Pb(II)	Absorption maximum ≈ 640 nm	PPD-modified PAN fibre	≈5.8	Cmin = 0.24 µg L^−1^	Immobilised on PPD matrix; performance reported for sorption–spectroscopic determination	[18]
PAR	Hg(II), Pb(II), Cd(II), Zn(II), Ni(II), Cu(II)	Visible colour response; red–orange → dark brown	Aminated PAN fibre	Neutral aqueous medium; selectivity increases at lower pH	Visual Pb(II) detection limit = 1 × 10^−6^ mol L^−1^	Covalent immobilisation via Mannich reaction; >50 reuse cycles; >30 days photostability	[38]
Dithizone	Hg(II)	Solid-phase colourimetric response	PANV-AV-17 fibrous support	0.2 mol L^−1^ NaCl medium	LOD = 0.01 mg L^−1^	Used on a dedicated PAN fibre disc in a sequential Hg/Cd/Pb procedure; long-term storage data n.r.	[39]
Xylenol orange	Bi(III)	Absorption ≈530 nm; reflectance ≈550 nm	Modified PAN fibre (PPM-1)	1.37	Detection limit = 0.12 μg mL^−1^; formation time = 10 min	Immobilisation improved response and reduced interference from Cu(II), Ag(I), Pd(II), and Fe(III)	[40]
DHNDSADNa	Cr(VI)	460 nm (immobilised reagent); 590 nm (Cr(VI) system)	Polyethylene-polyamine-modified PAN (PPA)	Study-specific	Detection limit = 2 μg mL^−1^	Rapid, selective, and regenerable immobilised reagent	[41]
AMADA	Mn(II)	500 nm (immobilised reagent); 580 nm (Mn(II) complex)	Polyethylene-polyamine-modified PAN (PPP)	Study-specific	Determination limit = 0.02 μg mL^−1^	Immobilised reagent showed high selectivity and suitability for repeated analytical use	[42]

Note: Reported optical and stability data are system-specific and should not be transferred between supports. DHNDSADNa = 4,5-dihydroxynaphthalene-2,7-disulphonic acid disodium salt; AMADA = alizarin-3-methylamino-N,N-diacetic acid; PPA = polyethylenepolyamine-modified PAN; Xylenol orange/Bi(III) is included for completeness of the PAN-immobilised reagent survey despite Bi(III) falling outside the primary target-metal scope defined in Table 1.

**Table 4:** Consolidated analytical figures of merit for SSA of toxic metal ions on fibrous and polymer supports (literature 2015–2026). All LOD values refer to the solid-phase procedure; solution spectrophotometric LODs cited in parentheses where available for comparison.

Metal	Sorbent/Carrier	Reagent/Detection Mode	pH	λ, nm	LOD/ Determination Limit	Linear Range	Ref.
Pb(II)	PPD (PAN/PEPA/DCE)	Arsenazo III/sorption–spectrophotometry	5.8	640	0.24 μg L^−1^	0.04–2.4 μg mL^−1^	[18]
Hg(II)	PANV-AV-17	Dithizone/solid-phase spectrophotometry	—	—	0.01 mg L^−1^	NR	[39]
Cd(II)	PANV-AV-17	PAR/solid-phase spectrophotometry	—	—	0.02 mg L^−1^	NR	[39]
Pb(II)	PANV-AV-17	PAR/solid-phase spectrophotometry	—	—	0.02 mg L^−1^	NR	[39]
Cu(II)	PANV-KU-2-PAN	1-(2-pyridylazo)-2-naphthol/diffuse reflectance	acidic	640	0.03 μg mL^−1^	0.05–0.40 μg mL^−1^	[29]
Cr(VI)	PANV-AV-17/PANV-KU-2 two-layer fibre	1,5-Diphenylcarbazide/diffuse reflectance	—	—	0.002 mg mL^−1^	0.002–0.015 mg mL^−1^	[63]
Ni(II)	PANV-KU-2	Dimethylglyoxime/diffuse reflectance	—	—	0.02 mg mL^−1^	0.02–0.10 mg mL^−1^	[63]
Cu(II)	PANV-KU-2	Sodium diethyldithiocarbamate/diffuse reflectance	—	—	0.02 mg mL^−1^	0.02–0.15 mg mL^−1^	[63]
Co(II)	Cation-exchange fibre	UV–Vis + PLS	optimised	400–700	0.15 μg L^−1^	NR	[64]
Ni(II)	Cation-exchange fibre	UV–Vis + PLS	optimised	400–700	0.13 μg L^−1^	NR	[64]
Cu(II)	Cation-exchange fibre	UV–Vis + PLS	optimised	400–700	0.10 μg L^−1^	NR	[64]
Mn(II)	PPP (PEPA-modified PAN)	*AMADA*/spectrophotometry	study-specific	580	0.02 μg mL^−1^	NR	[42]
Bi(III)	PPM-1 modified PAN fibre	Xylenol Orange/absorption–diffuse reflectance	1.37	530/550	0.12 μg mL^−1^	3–42 μg/25 mL	[40]

Note: NR = not reported; PLS = partial least squares; PANV-AV-17 and PANV-KU-2 = fibrous polyacrylonitrile-based sorbents; AMADA = alizarin-3-methylamino-N,N-diacetic acid; PPD = PAN/polyethylenepolyamine/1,2-dichloroethane; LOD or determination-limit values are reported as given in the original publications; where only a working concentration range was reported, the value is not interpreted as a conventional LOD.

**Table 5:** Matrix-specific validation and representative analytical performance of sorption–spectroscopic and related solid-phase methods.

Matrix/Sample Type	Analytical System	Analyte(s)	Analytical Performance	Validation/Comparison	Ref.
Water samples/model mixtures	PPD–Arsenazo III sorption–spectrophotometry	Pb(II)	Cmin = 0.24 μg L^−1^; Sr = 0.06; λmax = 640 nm; pH 5.8; linear range = 0.04–2.4 μg mL^−1^	Student’s *t*- and Fisher’s *F*-tests; direct determination in water samples	[18]
Industrial/real samples	AMADA immobilised on polyethylene-polyamine-modified PAN (AMADA/PPP)	Mn(II)	Determination limit = 0.02 μg mL^−1^ (20 μg L^−1^); analytical signals at 500 and 580 nm	Spectrophotometry, potentiodynamic titration, XRF, and selectivity studies	[42]
Water/environmental samples	DHNDSADNa immobilised on PPA	Cr(VI)	LOD = 2 μg mL^−1^; λ = 460 nm (reagent) and 590 nm (Cr(VI) complex)	Spectroscopic/X-ray fluorescence and potentiodynamic titration; interference study	[41]
Water, wastewater, and tea infusion	MnSb_2_O_6_@Fe_3_O_4_ magnetic SPE–FAAS	Cu(II)	LOD = 2.1 ng mL^−1^; linear range = 10–200 ng mL^−1^; recovery = 93–102%; 48-fold sensitivity enhancement	Applied to wastewater, tap water, and apple-tea samples	[85]
Soil and water CRMs	ICP-based elemental analysis	Cr, Mn, Cu, As, Cd, Ba, Hg, Pb	CRM recovery = 93.2–107.6%	SRM 2709a (San Joaquin soil) and SRM 1640a (water)	[99]
Sewage sludge/ERM-CC144	Conventional and ultrasound-assisted BCR sequential extraction + ICP-OES/CVAAS	Cd, Cr, Cu, Ni, Pb, Zn, Hg	ERM-CC144 recoveries: Cd 87.4%, Cr 90.0%, Cu 101.0%, Ni 91.8%, Pb 96.6%, Zn 94.7%, Hg 87.4%; RSD 0.3–2.9%	ERM-CC144 certified reference material; conventional vs. ultrasound-assisted extraction	[100]
Food and water	PvbXa-based vortex-assisted dispersive SPE–FAAS	Cd(II)	LOD = 0.06 μg L^−1^; linear range = 0.20–150 μg L^−1^; RSD = 4.3%; recovery in spiked water = 92–98%	Food/water application; multivariate optimisation	[72]
Cosmetic products	MWCNT-SPE–ICP-AES	As, Bi, Cd, Hg, Pb, Ti	LOD = 2.4, 4.08, 0.3, 1.8, 2.1 and 1.8 ng mL^−1^, respectively; RSD < 2.0%; recovery = 89.6–104.4%	NIST SRM 1570a spinach leaves; 34 cosmetic samples	[101]

Note: CRM = certified reference material; The SRM 2709a and ERM-CC144 entries represent independent CRM/reference-method benchmarks and should not be interpreted as SSA-specific validation data.

**Table 6:** Estimated AGREE subscores for batch sorption–spectroscopic analysis (SSA) using fibrous PAN sorbents compared with chloroform-based liquid–liquid extraction (LLE) and electrothermal atomic absorption spectrometry (ETAAS) following coprecipitation. Scores were estimated from reported procedure characteristics according to the AGREE framework [13,102] and are intended as comparative estimates rather than experimentally measured environmental-impact values. Higher scores indicate more favourable performance for the corresponding GAC principle.

GAC Principle	PAN-Based Batch SSA	LLE/CHCl_3_	ETAAS/ Co-Precipitation
P1. Direct analytical measurements/minimise sample preparation	0.8	0.4	0.5
P2. Minimise sample size and number of analytical steps	0.7	0.2	0.3
P3. Avoid hazardous chemicals and reagents	0.8	0.0	0.2
P4. Prefer renewable reagents and materials	0.3	0.1	0.2
P5. Minimise waste generation	0.7	0.2	0.4
P6. Avoid unnecessary derivatisation	0.8	0.5	0.5
P7. Miniaturise and reduce analytical scale	0.8	0.2	0.5
P8. Integrate analytical operations	0.9	0.3	0.4
P9. Prefer multi-analyte methods where applicable	0.6	0.4	0.3
P10. Reduce energy consumption	0.8	0.5	0.2
P11. Reduce occupational hazards	0.8	0.1	0.4
P12. Promote safe and sustainable analytical procedures	0.8	0.2	0.4
Overall assessment	Favourable	Less favourable	Intermediate

## Data Availability

No new experimental data are reported in this review. All data discussed are available in the primary publications listed in References.

## Supplementary Materials

The following supporting information can be downloaded at: https://www.mdpi.com/article/10.3390/polym18182218/s1, Figure S1: Literature-screening overview of the Scopus corpus, including annual publication trends, the historical publication timeline, and explicit AGREE/GAPI reporting within the screened literature corpus.

## Abbreviations

The following abbreviations are used in this manuscript:

AAS	atomic absorption spectrometry
AGREE	Analytical GREEnness metric
AMADA	alizarin-3-methylamino-N,N-diacetic acid
AOPAN	amidoximated polyacrylonitrile
CRM	certified reference material
DCE	1,2-dichloroethane
DFT	density functional theory
DHNDSADNa	4,5-dihydroxynaphthalene-2,7-disulphonic acid disodium salt
ETAAS	electrothermal atomic absorption spectrometry
FAAS	flame atomic absorption spectrometry
FIA	flow-injection analysis
FIBAN	fibrous ion-exchange material
GAPI	green analytical procedure index
HSAB	hard and soft acids and bases
ICP-MS	inductively coupled plasma mass spectrometry
ICP-OES	inductively coupled plasma optical emission spectrometry
LOD	limit of detection
LOQ	limit of quantification
LOV	lab-on-valve
MAC	maximum admissible concentration
MCL	maximum contaminant level
MSPE	magnetic solid-phase extraction
PAN	polyacrylonitrile
PAN-T	triethanolamine-modified polyacrylonitrile
PAR	4-(2-pyridylazo)resorcinol
PEPA	polyethylenepolyamine
PPA	PAN/polyethylenepolyamine/acrylonitrile matrix
PPD	PAN/polyethylenepolyamine/1,2-dichloroethane matrix
RSD	relative standard deviation
SIA	sequential injection analysis
SPE	solid-phase extraction
SSA	sorption–spectroscopic analysis
SRM	standard reference material
UV–Vis	ultraviolet–visible spectroscopy
XO	xylenol orange
